# Advancements and Prospects in Nanorobotic Applications for Ophthalmic Therapy

**DOI:** 10.1021/acsbiomaterials.4c02368

**Published:** 2025-01-17

**Authors:** Antonio Átila Menezes Ferreira, John Hebert da Silva Felix, Rita Karolinny Chaves de Lima, Maria Cristiane Martins de Souza, José Cleiton Sousa dos Santos

**Affiliations:** Instituto de Engenharias e Desenvolvimento Sustentável, Universidade da Integração Internacional da Lusofonia Afro-Brasileira, Campus das Auroras, Redenção, Ceará CEP 62790-970, Brazil

**Keywords:** nanotechnology, glaucoma, drug delivery, bibliometric analysis

## Abstract

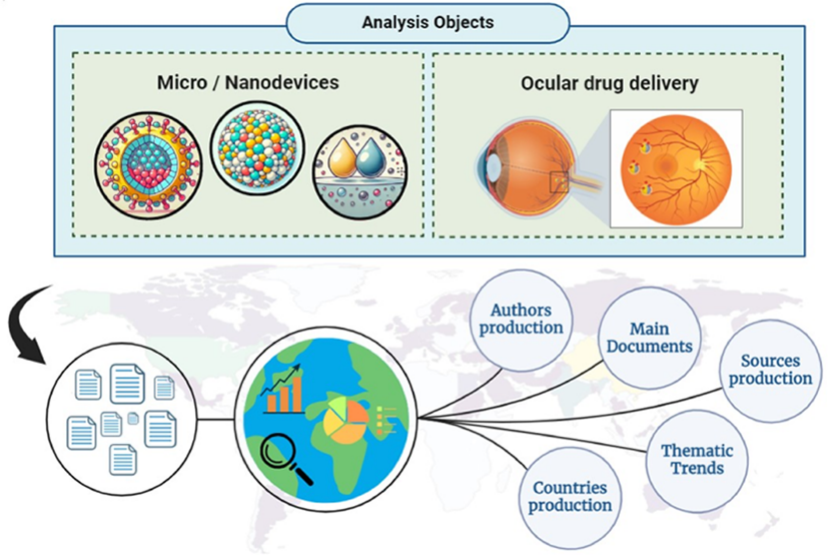

This study provides a bibliometric and bibliographic review of emerging applications of micro- and nanotechnology in treating ocular diseases, with a primary focus on glaucoma. We aim to identify key research trends and analyze advancements in devices and drug delivery systems for ocular treatments. The methodology involved analyzing 385 documents indexed on the Web of Science using tools such as VOSviewer and Bibliometrix. The results show a marked increase in scientific output, highlighting prominent authors and institutions, with England leading in the field. Key findings suggest that nanotechnology holds the potential to address the limitations of conventional treatments, including low ocular bioavailability and adverse side effects. Nanoparticles, nanovesicles, and polymer-based systems appear promising for prolonged and controlled drug release, potentially offering enhanced therapeutic efficacy. In conclusion, micro- and nanotechnology could transform ocular disease treatment, although challenges remain concerning the biocompatibility and scalability of these devices. Further clinical studies are necessary to establish these innovations within the therapeutic context of ophthalmology.

## 1 Introduction

Ocular diseases are a significant global public health issue, impacting millions worldwide^1,2^ and often leading to serious consequences, such as irreversible blindness, even when treatments are available.^3^ Among these diseases, glaucoma is a leading cause of irreversible blindness, characterized by progressive optic neuropathy resulting in the loss of retinal ganglion cells.^4,5^ Significant forms of glaucoma include primary open-angle glaucoma (POAG), primary angle-closure glaucoma (PACG), and secondary glaucoma, each with distinct pathological mechanisms.^6^

POAG is marked by visual field loss due to poor drainage of aqueous humor and is associated with risk factors such as advanced age and elevated intraocular pressure (IOP).^7−9^ PACG occurs when the angle between the iris and cornea closes, blocking aqueous humor drainage.^10^ Secondary glaucoma also leads to increased IOP but results from preexisting eye conditions or injuries. Notable types include neovascular glaucoma,^11^ corticosteroid-induced glaucoma,^12^ and tumor-related secondary glaucoma.^13^ Out of these forms, POAG is the most prevalent worldwide.^14−16^

Conventional treatments for glaucoma primarily aim to lower IOP. The first line of treatment typically involves topical medications, such as prostaglandin analogues, beta-blockers, carbonic anhydrase inhibitors, and adrenergic agonists.^17−19^ When medications prove insufficient, alternatives such as laser trabeculoplasty and filtering surgeries may be employed to reduce IOP.^20−22^ However, these topical treatments are often accompanied by side effects, including hyperemia, eye irritation, keratitis, and uveitis.^23−25^ Patients who cannot regulate IOP with a single medication usually require multiple drugs, adding complexity to their treatment regimens.^26^ Additionally, while effective, laser trabeculoplasty may require repeated treatments over time, which increases the risk of complications.^20^

As an alternative to these conventional approaches, micro- and nanotechnology have emerged as promising solutions for glaucoma treatment, offering minimally invasive options with enhanced therapeutic efficacy. Microstents and micromachines allow for interventions that reduce IOP with fewer complications and less reliance on medication.^27,28^ Furthermore, nanotechnology-based treatment systems enhance bioavailability and ocular penetration, minimize side effects, and improve therapeutic outcomes.^29,30^

Nanoparticles enhance drug delivery by extending drug release and improving corneal permeability, resulting in more efficient ocular delivery. For example, chitosan nanoparticles loaded with betaxolol hydrochloride have significantly reduced IOP in experimental glaucoma models.^31^ Similarly, chimeric chitosan nanoparticles loaded with vancomycin have demonstrated increased ocular retention and penetration, effectively overcoming natural ocular barriers and providing sustained drug release for treating resistant bacterial infections.^32^ These advances are complemented by drug-loaded contact lens systems, offering prolonged and controlled drug delivery.^33^ Additionally, gene therapy is being explored for eye conditions like age-related macular degeneration and diabetic retinopathy by using noncoding RNAs, such as microRNA (miRNA) and small interfering RNA (siRNA).^34^ Nanotechnology also benefits other areas of medicine; for instance, nanoemulgels simultaneously deliver hydrophilic and lipophilic drugs, enhancing the safety and efficacy of treatments for conditions like facial rosacea.^35^

Microtechnology is also essential in developing minimally invasive devices for glaucoma treatment. For example, biodegradable PLGA microparticles are being studied for their therapeutic potential.^36^ Devices such as MicroShunt have shown promising outcomes in multicenter studies, achieving significant, sustained reductions in IOP in patients with primary open-angle glaucoma.^37^ They are being compared favorably to trabeculectomy.^38^ Implantable stents, such as the CyPass Micro-Stent, have also been studied with cataract surgery, demonstrating effective IOP reduction and decreased use of ocular hypotensive medications.^28^ Additionally, microrobots have been investigated for minimally invasive intraocular surgeries, showing promising mobility and precision within the ocular environment.^39^ Together, these advancements reduce the need for invasive procedures and offer more effective treatment options.^29,39^

Given the growing relevance of micro- and nanometric technologies in treating glaucoma, this review presents a detailed bibliometric analysis of the leading research indicators in the area. The study focuses on developing and applying micro- and nanodevices, exploring their contributions to ophthalmic therapy, especially in drug delivery systems. The analysis covers technological advances, innovation trends, and the combination of these devices with drugs to form delivery systems. Bibliometrics, a qualitative and quantitative analytical tool,^40−42^ evaluates and maps scientific output in specific fields,^43,44^ helping to measure the impact of publications, identify research trends, and assess scientific performance.^45,46^ This method also facilitates the analysis of collaborative networks and the identification of emerging areas across disciplines.^47−49^ The study aims to conduct a bibliometric and bibliographic analysis to identify prominent authors,^50−52^ institutions,^53,54^ topics,^55−57^ and subfields and evaluate practical applications,^58−60^ key patents,^61−63^ and various drug delivery systems.

## 2 Methodology

The bibliometric analysis was conducted using data from the Web of Science (WoS) database, yielding 385 articles spanning 10 years (2014–2024). Following data collection, analysis was performed using tools such as VOSviewer, the Biblioshiny interface in the Bibliometrix library, and custom plotting scripts developed in Python. These tools enabled the calculation of bibliometric indices, including h, m, and g indices, and the identification of keyword co-occurrence networks and associations among key authors,^64,65^ institutions,^66,67^ journals,^68^ and countries to better understand and analyze the data.

**Figure 1:**
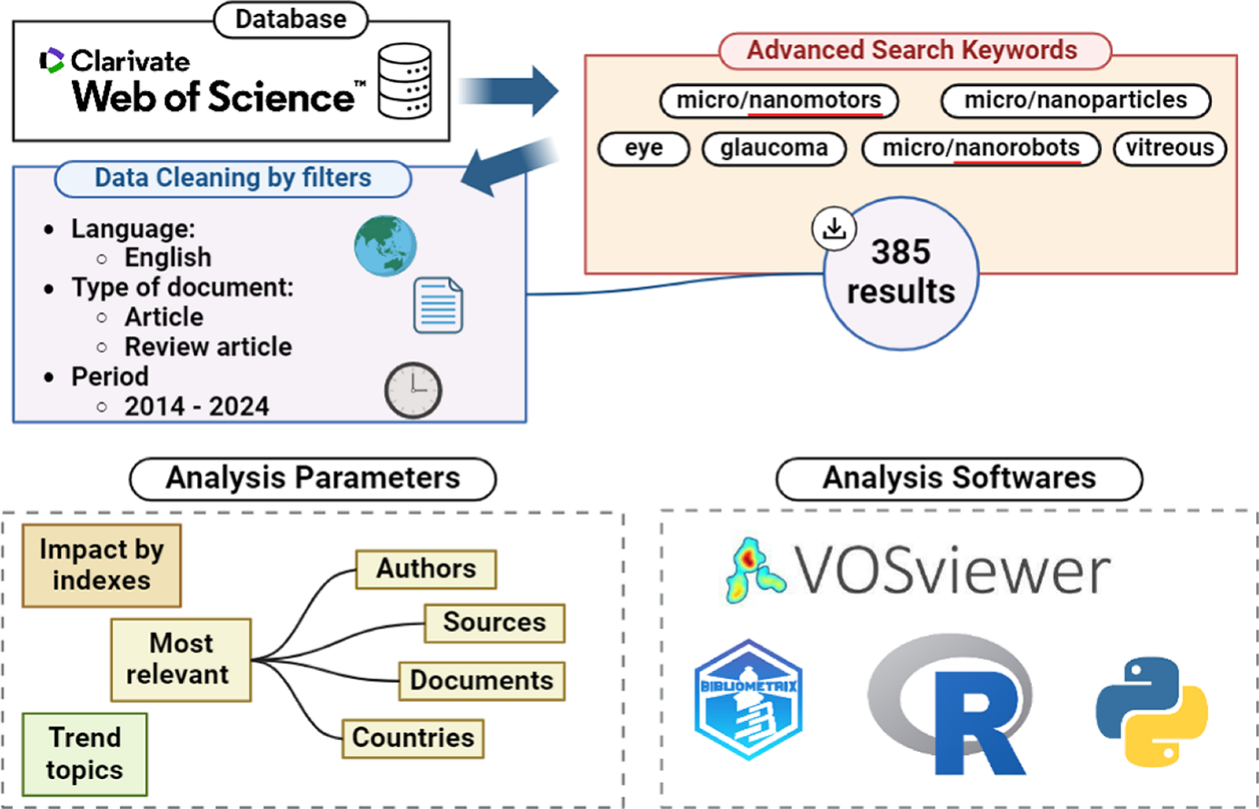
Methodological process of bibliometric analysis.

Data collection included keywords related to micro- and nanotechnology, and filters were applied based on the document type. Figure 1 illustrates the step-by-step process used to refine the data. For a comprehensive review, terms such as “micro” or “nanomachines,” “motors,” “particles,” and “robots,” as well as terms relevant to the study’s focus on eye disease and glaucoma, were used. Table 1 details the setup of the advanced search bar.

**Table 1:** Search Prompt and Results Obtained

Prompt	Results
((ALL = (vitreous) OR ALL = (eye)) AND (ALL = (glaucoma)) AND (ALL = (microrobot OR nanorobot) OR ALL = (micromachine OR nanomachine) OR ALL = (micromotor OR nanomotor) OR ALL = (microparticle OR nanoparticles)))	385 documents

In addition, it was necessary to normalize the data related to the production and citations of countries, taking into account that countries with larger populations, such as China, naturally tend to have more publications. Therefore, to normalize the data, eqs 1 and 2 were implemented.
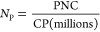
1

Note: NP = normalized publications; PNC = publication number per country; CP = country population.
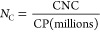
2

Note: N_P_ = normalized citations; CNC = citation number per country; CP = country population.

## 3 Results and Discussion

This section presents the main findings of the bibliometric analysis, organized into five subtopics: productivity analysis (by author, journal, and country), document analysis, thematic trend analysis (keywords and indexing terms), and a comprehensive critical review of each device and its application in glaucoma treatment.

### 3.1 Bibliometric Review

**Figure 2:**
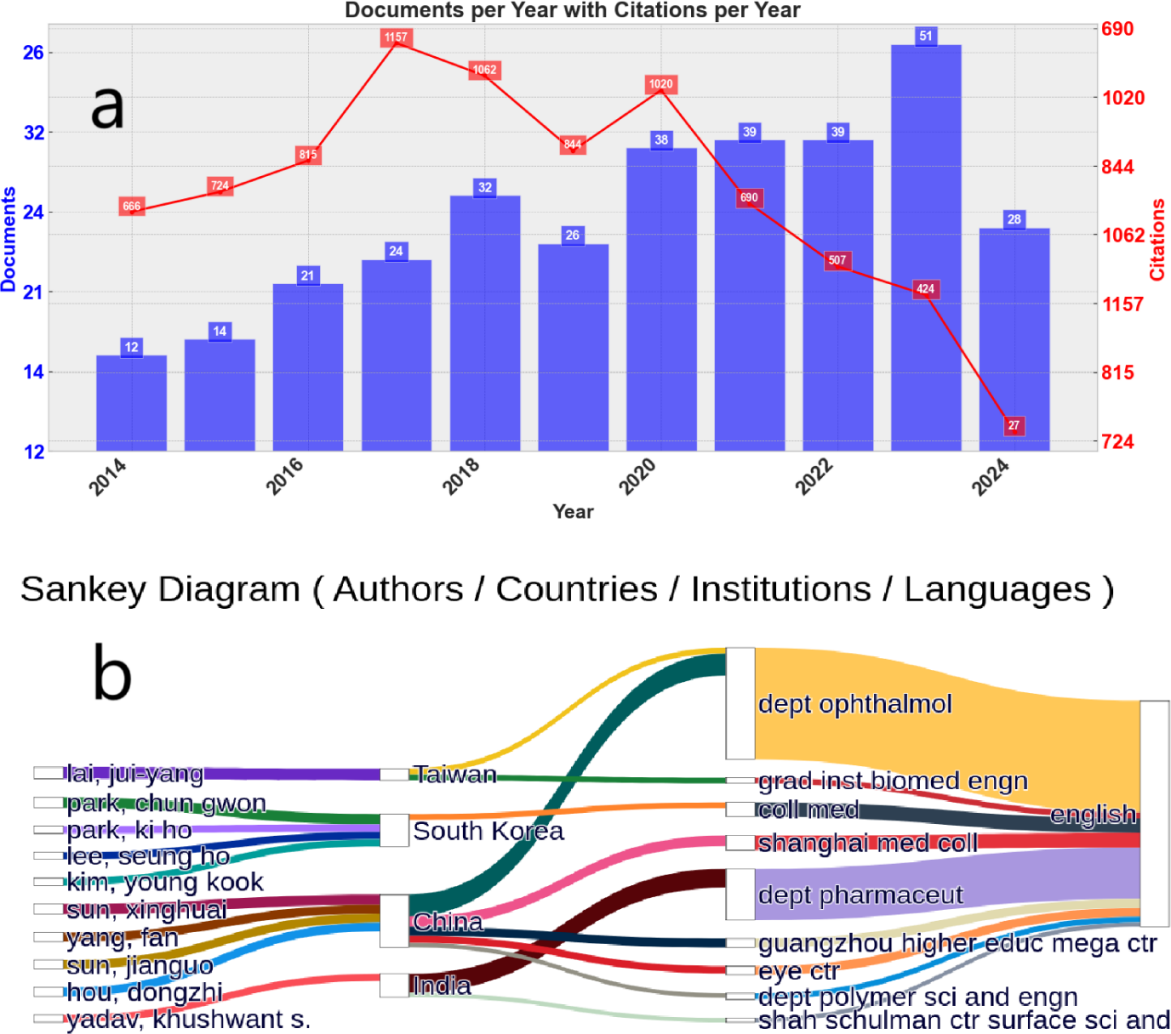
Graphical representation of the bibliometric data. (a) Annual number of documents and citations; (b) Sankey diagram illustrating correlations among authors, journals, countries, and languages.

To begin the bibliometric analysis, the leading information report in Table 2 provides general information about the bibliometric results. Figure 2a presents a quantitative visualization of the scientific output over time, showing an increase in publications on this topic and suggesting that this is a promising field with the potential to change the perspectives of glaucoma treatment. In addition, Figure 2b shows a correlation diagram linking key analysis indicators (authors, countries, and journals), providing an overview of the relationships within this research landscape.

**Table 2:** Main Report on Information

Main information	Results
timespan	2014–2024
total number of countries	51
total number of institutions	525
total number of sources	147
total number of references	16813
total number of languages	1
--English (# of docs)	324
total number of documents	324
--Article	246
--Review	78
average documents per author	1.21
average documents per institution	4.73
average documents per source	2.2
average documents per year	29.45
total number of authors	1687
total number of authors keywords	905
total number of authors keywords plus	1095
total single-authored documents	1
total multi-authored documents	323
average collaboration index	6.25
max h-index	7
total number of citations	7936
average citations per author	4.7
average citations per institution	15.12
average citations per document	24.49
average citations per source	53.99

#### 3.1.1 Authors

**Figure 3:**
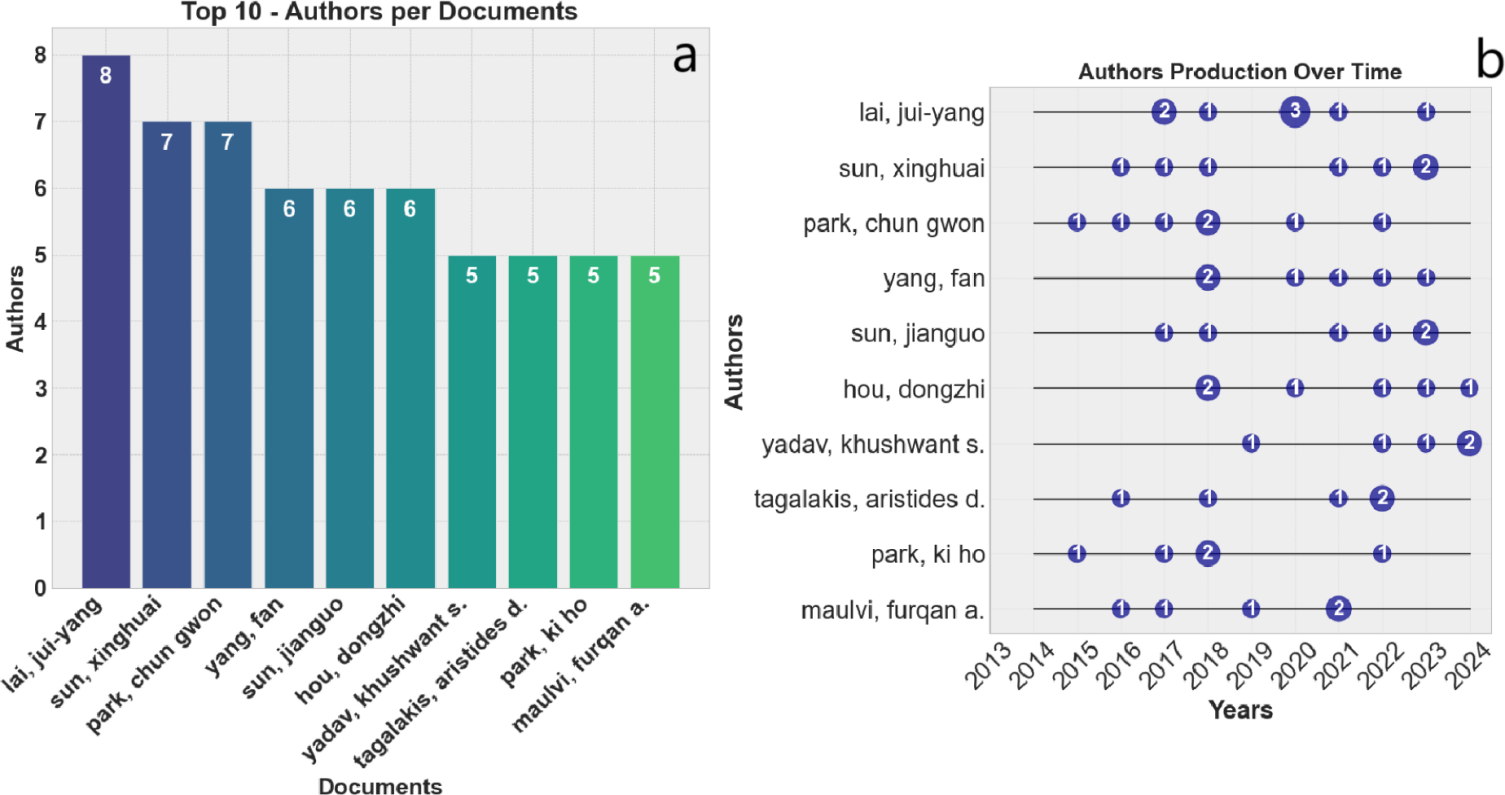
Analysis of author productivity. (a) Top 10 authors by total documents produced; (b) annual distribution of publications by the 10 most prolific authors.

**Figure 4:**
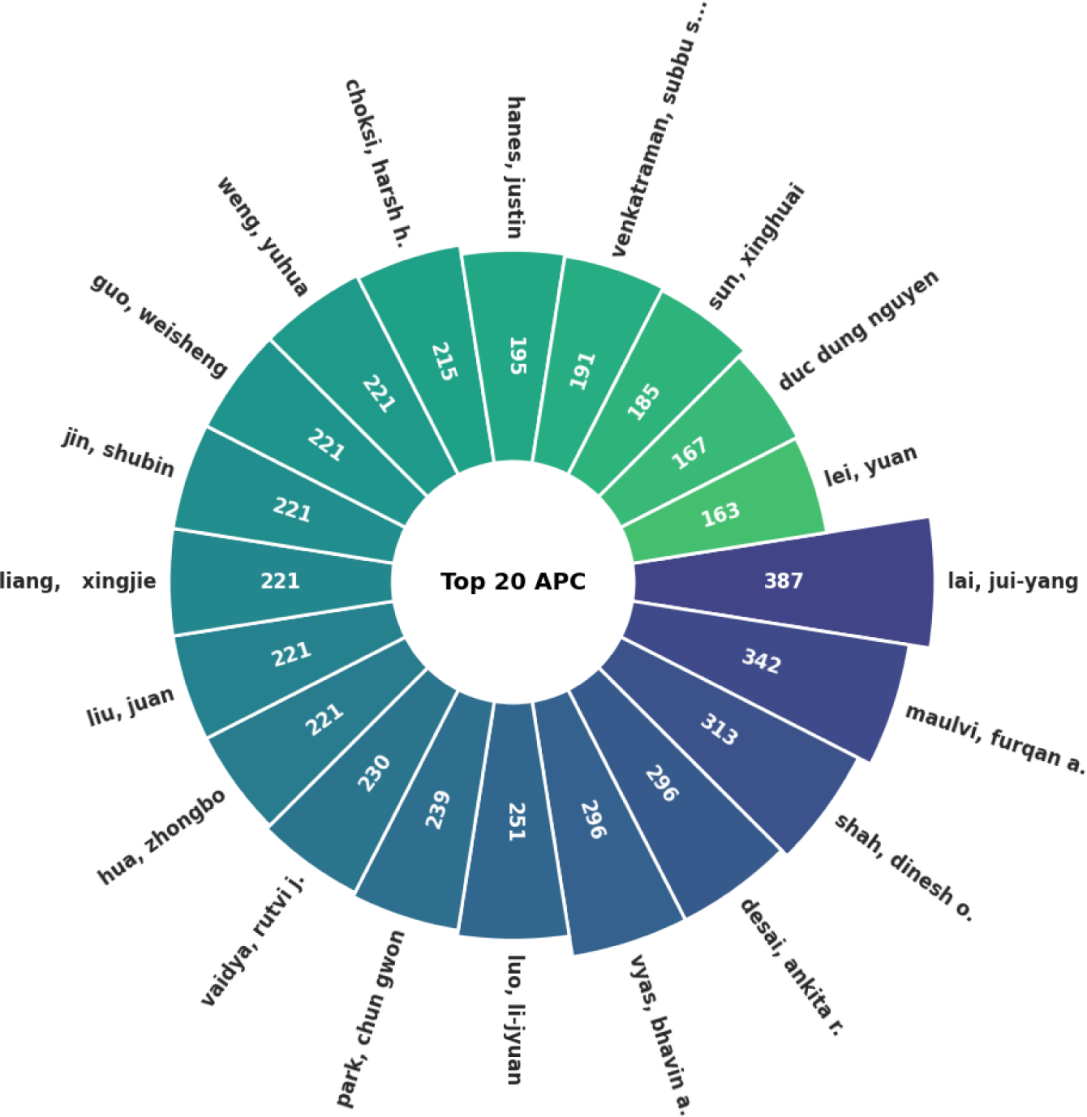
Circular bar chart of the top 20 most cited authors.

This study examined academic productivity in the field of micro- and nanotechnology applications for glaucoma treatment, identifying a total of 1687 authors. Figures 3a,b displays the most prolific authors over the years, while Figure 4 highlights those with the highest citation counts.

Table 3 presents the recent contributions of each author, showcasing various ocular drug delivery devices, including nano eye drops,^69^ hydrogels,^70^ and intraocular delivery systems,^71^ as well as controlled-release devices.^72^ The central focus of these articles is the development of advanced drug delivery systems for ocular applications, emphasizing improved bioavailability, targeted delivery, and innovative technologies to enhance treatment efficacy. This focus is evident in the recent work of the most prolific author, Jui-Yang Lai, who has explored methods such as amine-mediated eye drops to enhance corneal permeability,^69^ multifunctional hydrogels responsive to glutathione levels,^70^ and cerium nanoparticles with tunable cavities.^73^

**Table 3:** Top Publications by the Most Relevant Authors

**n**	**Author**	**Year**	**Title**	**Source**	**ref.**
1	Lai, Jui-Yang	2023	Amination-mediated nano eye-drops with enhanced corneal permeability and effective burst release for acute glaucoma treatment	CHEMICAL ENGINEERING JOURNAL	(69)
		2021	Harnessing the tunable cavity of nanoceria for enhancing Y-27632-mediated alleviation of ocular hypertension	THERANOSTICS	(73)
		2020	Multifunctional glutathione-dependent hydrogel eye drops with enhanced drug bioavailability for glaucoma therapy	CHEMICAL ENGINEERING JOURNAL	(70)
2	Sun, Xinghuai	2023	Intraocular nanomicroscale drug delivery systems for glaucoma treatment: design strategies and recent progress	JOURNAL OF NANOBIOTECHNOLOGY	(78)
		2023	Trans-corneal drug delivery strategies in the treatment of ocular diseases	ADVANCED DRUG DELIVERY REVIEWS	(79)
		2022	Sustained release of brimonidine from BRI@SR@TPU implant for treatment of glaucoma	DRUG DELIVERY	(71)
3	Park, Chun Gwon	2022	Iontophoretic ocular delivery of latanoprost-loaded nanoparticles via skin-attached electrodes	ACTA BIOMATERIALIA	(80)
		2020	Brimonidine-montmorillonite hybrid formulation for topical drug delivery to the eye	JOURNAL OF MATERIALS CHEMISTRY B	(81)
		2018	Amino-Functionalized Mesoporous Silica Particles for Ocular Delivery of Brimonidine	MOLECULAR PHARMACEUTICS	(82)
4	Yang, Fan	2023	Physicochemical properties and microinteraction between micronanoparticles and anterior corneal multilayer biological interface film for improving drug delivery efficacy: the transformation of tear film turnover mode	DRUG DELIVERY	(83)
		2022	Critical Evaluation of Multifunctional Betaxolol Hydrochloride Nanoformulations for Effective Sustained Intraocular Pressure Reduction	INTERNATIONAL JOURNAL OF NANOMEDICINE	(84)
		2021	Microinteraction of mucin tear film interface with particles: the inconsistency of pharmacodynamics and precorneal retention of ion-exchange, functionalized, Mt-embedded nano- and microparticles	COLLOIDS AND SURFACES B-BIOINTERFACES	(85)
5	Sun, Jianguo	2023	Intraocular nanomicroscale drug delivery systems for glaucoma treatment: design strategies and recent progress	JOURNAL OF NANOBIOTECHNOLOGY	(78)
		2023	A Dual-Drug Nanohybrid System Incorporating Nimodipine and Brain-Derived Neurotrophic Factor Promotes Retinal Ganglion Cells Survival	ADVANCED THERAPEUTICS	(86)
		2022	Sustained release of brimonidine from BRI@SR@TPU implant for treatment of glaucoma	DRUG DELIVERY	(71)
6	Hou, Dongzhi	2024	Microinteraction of montmorillonite-loaded nanoparticles with mucin promotes retention of betaxolol hydrochloride on the ocular surface and the tear film microenvironment	APPLIED CLAY SCIENCE	(87)
		2023	Physicochemical properties and microinteraction between micronanoparticles and anterior corneal multilayer biological interface film for improving drug delivery efficacy: the transformation of tear film turnover mode	DRUG DELIVERY	(83)
		2022	Critical Evaluation of Multifunctional Betaxolol Hydrochloride Nanoformulations for Effective Sustained Intraocular Pressure Reduction	INTERNATIONAL JOURNAL OF NANOMEDICINE	(84)
7	Yadav, Khushwant S.	2024	Advances in drug-loaded contact lenses for glaucoma: materials, evaluation parameters, and novel drug delivery strategies with modified nanoparticles	JOURNAL OF DRUG DELIVERY SCIENCE AND TECHNOLOGY	(72)
		2024	Development of brimonidine niosomes laden contact lenses for extended release and promising delivery system in glaucoma treatment	DARU-JOURNAL OF PHARMACEUTICAL SCIENCES	(88)
		2023	Bimatoprost-loaded lipidic nanoformulation development using quality by design: liposomes versus solid lipid nanoparticles in intraocular pressure reduction	NANOMEDICINE	(89)
8	Tagalakis, Aristides D.	2022	Non-Viral Gene Therapy in Trabecular Meshwork Cells to Prevent Fibrosis in Minimally Invasive Glaucoma Surgery	PHARMACEUTICS	(90)
		2022	Advances in exosome therapies in ophthalmology-From bench to clinical trial	ACTA OPHTHALMOLOGICA	(91)
		2021	Novel PEGylated Lipid Nanoparticles Have a High Encapsulation Efficiency and Effectively Deliver MRTF-B siRNA in Conjunctival Fibroblasts	PHARMACEUTICS	(92)
9	Park, Ki Ho	2022	Iontophoretic ocular delivery of latanoprost-loaded nanoparticles via skin-attached electrodes	ACTA BIOMATERIALIA	(80)
		2018	Amino-Functionalized Mesoporous Silica Particles for Ocular Delivery of Brimonidine	MOLECULAR PHARMACEUTICS	(82)
		2018	Metal–organic frameworks, NH < sub >2</sub>-MIL-88(Fe), as carriers for ophthalmic delivery of brimonidine	ACTA BIOMATERIALIA	(93)
10	Maulvi, Furqan A.	2021	Advances and challenges in the nanoparticles-laden contact lenses for ocular drug delivery	INTERNATIONAL JOURNAL OF PHARMACEUTICS	(94)
		2021	Controlled bimatoprost release from graphene oxide laden contact lenses: in vitro and in vivo studies	COLLOIDS AND SURFACES B-BIOINTERFACES	(95)
		2019	Effect of gold nanoparticles on timolol uptake and its release kinetics from contact lenses: in vitro and in vivo evaluation	ACTA BIOMATERIALIA	(96)

In addition to these studies, other research by Lai et al. delves into novel drug release mechanisms. Their work includes investigations on biodegradable poly(ε-caprolactone) nanocapsules for prolonged therapeutic effects,^74^ studies on the impact of poly(lactide) nanoparticle shell thickness on drug release rates, demonstrating that thicker shells slow capsule degradation,^75^ and the development of hollow cerium nanoparticles with dual antioxidant and anti-inflammatory functionality.^76^ Lai has also examined the release properties of chitosan polymers, showing that increased deacetylation enhances biodegradation resistance, resulting in a slower drug release.^77^

#### 3.1.2 Countries

**Figure 5:**
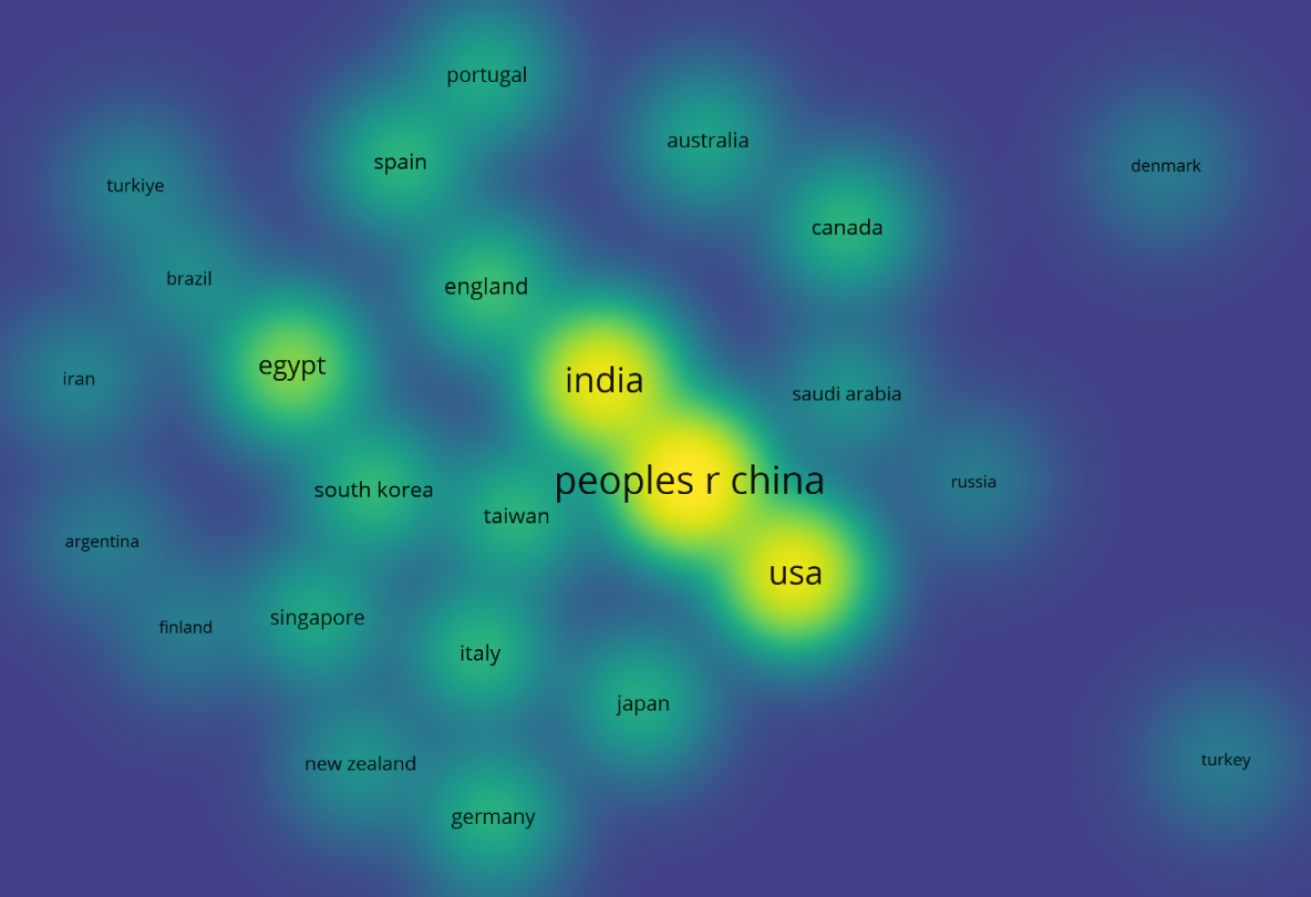
Heat map of document production by country. The size of each nucleus indicates the volume of documents produced, with larger nuclei representing higher output.

An analysis of scientific output by country reveals China’s dominance in publication volume and citation counts (Table 4 and Figure 5). This can be attributed to China’s extensive research infrastructure, significant government investment, and collaboration with foreign scientists.^97^ However, although China initially leads the ranking, the normalization of these metrics reveals a more balanced scenario if we consider the population’s size. Countries with smaller populations (Table 5), such as England and Taiwan, demonstrate significant productivity and a large volume of citations (Table 6) compared to their population size, which indicates high efficiency in research production. Normalization factors were obtained using eqs 1 and 2 presented earlier; the population quantity data were taken from the Population Pyramid^98^ and the Trading Economics^99^ websites.

**Table 4:** Scientific Production of Each Country

Country	No. of publications	No. of citations
China	81	1931
India	61	1258
USA	59	1660
Egypt	28	609
England	16	504
South Korea	14	436
Taiwan	13	515
Canada	13	382
Italy	13	347

**Table 5:** Central Countries and Population Quantity

Country	Country population (in millions)
China	14 225
India	1438
USA	3434
Egypt	1145
England	549
South Korea	517
Taiwan	2342
Canada	3929
Italy	5949

**Table 6:** Normalized Scientific Production and Citations are Given for Each Country

Country	Normalized publications	Normalized citations
England	29 143 898	9 180 327 869
Taiwan	0 555 081 127	2 198 975 235
Canada	0 330 872 996	9 722 575 719
South Korea	0 270 793 037	8 433 268 859
Egypt	0 244 541 485	5 318 777 293
Italy	0 218 524 122	5 832 913 095
USA	0 171 811 299	4 834 012 813
China	0 056 942 004	1 357 469 244
India	0 042 420 028	0 874 826 147

**Figure 6:**
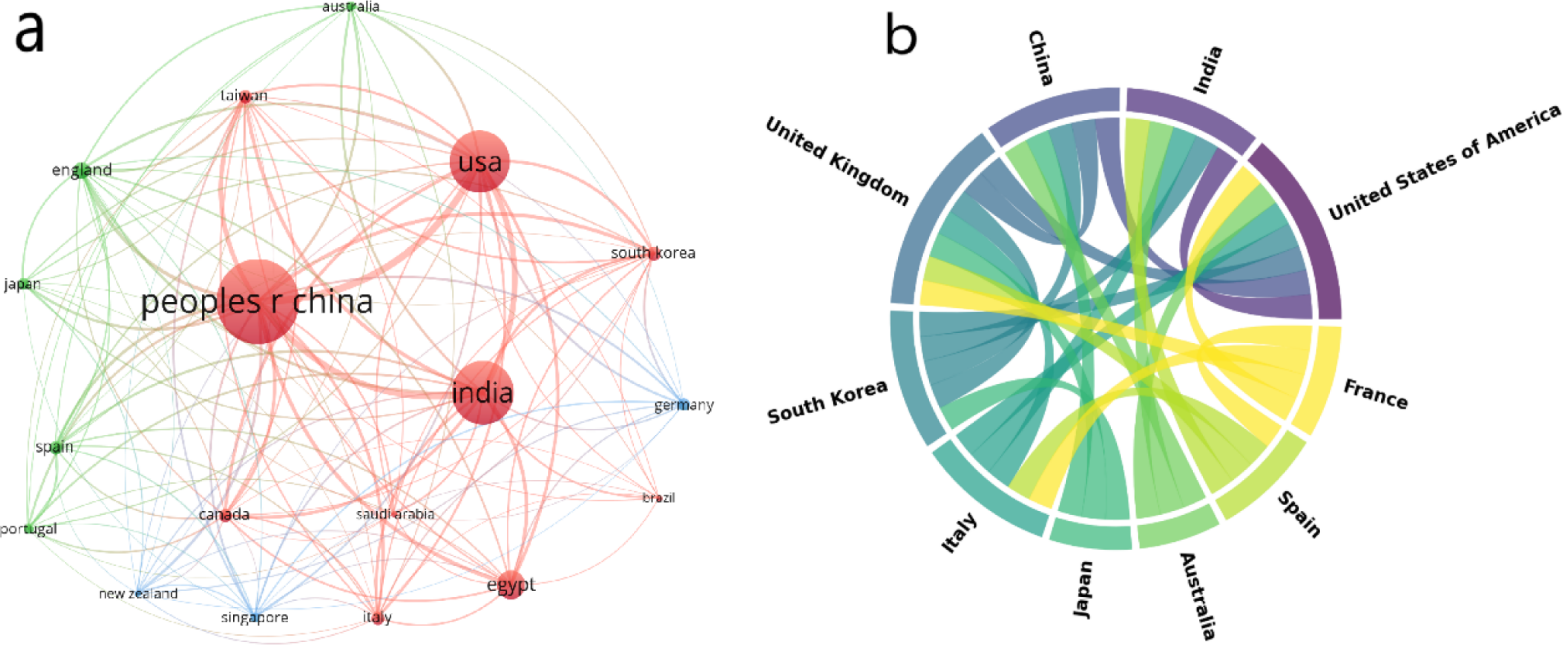
Cross-country collaboration networks. (a) Neural network illustrating cross-country collaborations; (b) chord diagram displaying organized collaboration links among countries.

International collaboration is critical in generating structured knowledge (Figure 6a). The plotted graphs show that collaborations across regions form strong links, with the United States connected to over 70% of these networks (Figure 6b).

#### 3.1.3 Journals

**Figure 7:**
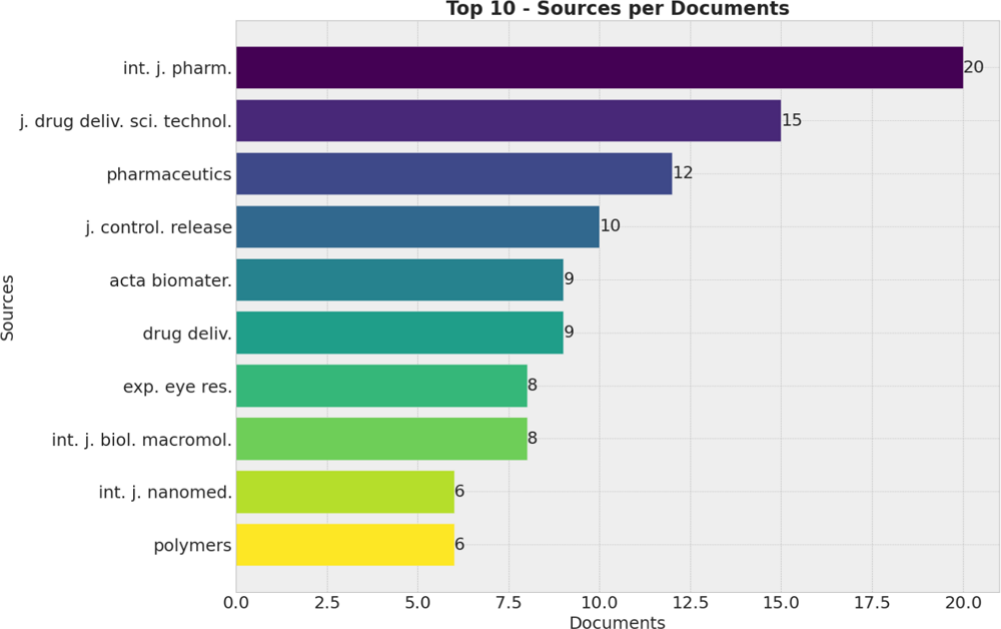
Number of publications per journal.

**Figure 8:**
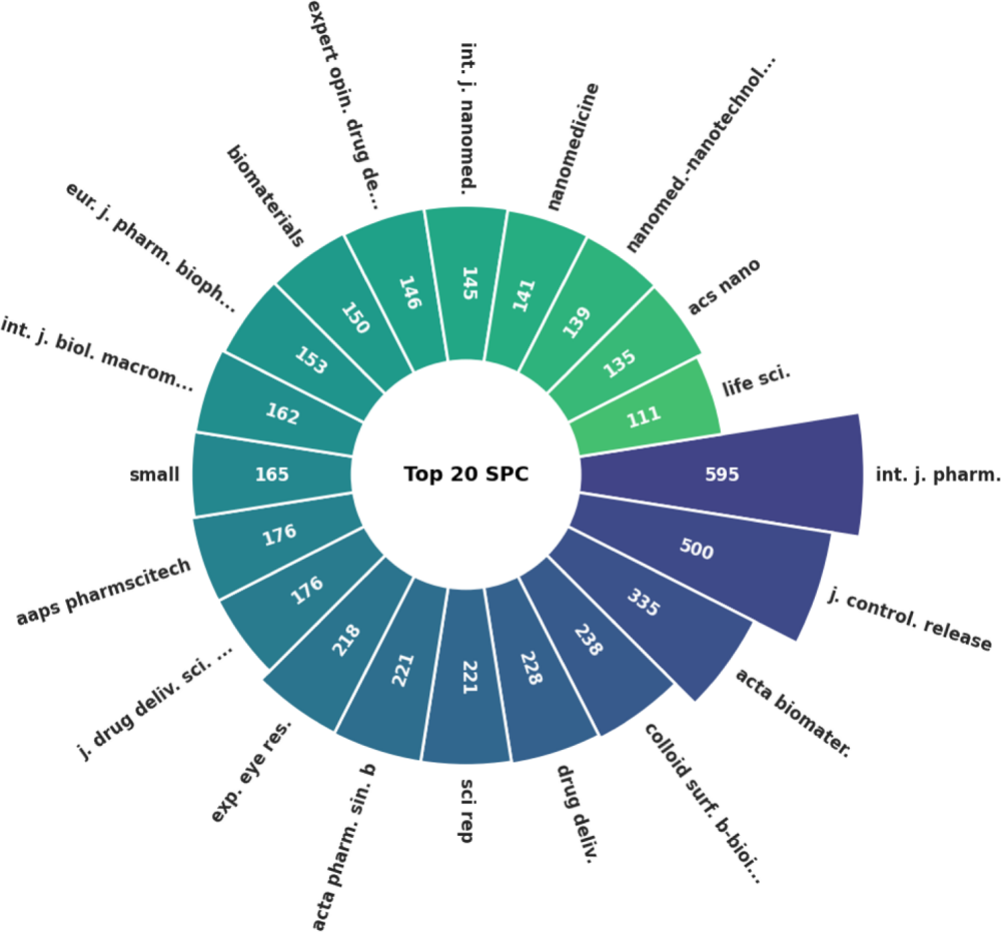
Number of sources per citation.

**Figure 9:**
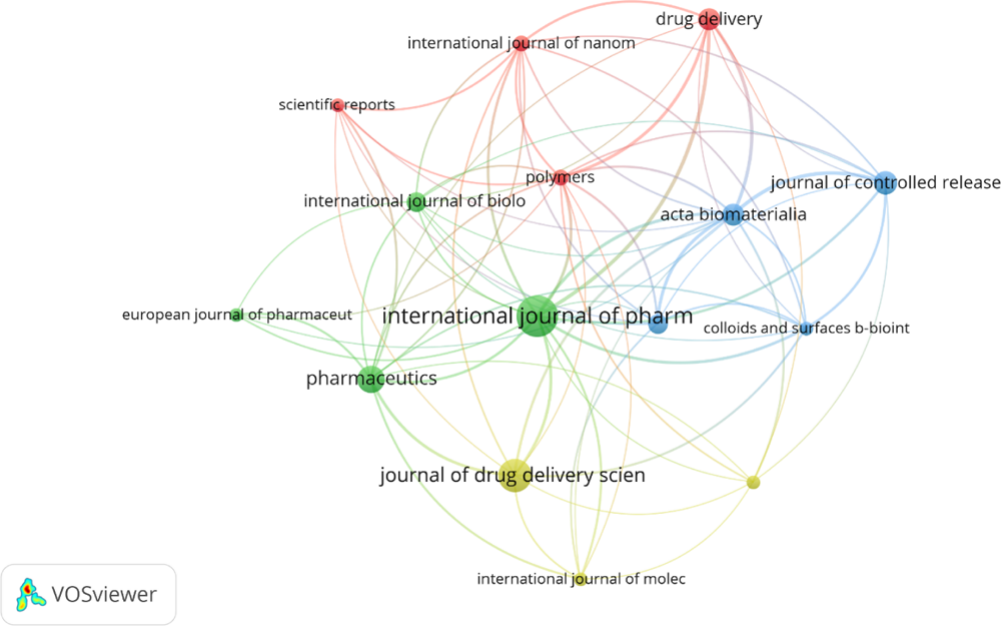
Neural network collaboration among leading journals.

In addition to authors and countries, the bibliometric analysis identified 147 scientific journals publishing on the application of micro- and nanotechnology in glaucoma treatment. Tables 7 and 8 and Figures 7 and 8 highlight the top 10 journals in terms of publication volume and citation count, respectively. Figure 9 displays the collaboration networks among these journals.

**Table 7:** Number of Documents per Journal

Source	No. of documents
INTERNATIONAL JOURNAL OF PHARMACEUTICS	20
JOURNAL OF DRUG DELIVERY SCIENCE AND TECHNOLOGY	15
PHARMACEUTICS	12
JOURNAL OF CONTROLLED RELEASE	10
ACTA BIOMATERIALIA	9
DRUG DELIVERY	9
EXPERIMENTAL EYE RESEARCH	8
INTERNATIONAL JOURNAL OF BIOLOGICAL MACROMOLECULES	8
POLYMERS	6
INTERNATIONAL JOURNAL OF NANOMEDICINE	6

**Table 8:** Most Cited Journals

Source	No. of citations
INTERNATIONAL JOURNAL OF PHARMACEUTICS	595
JOURNAL OF CONTROLLED RELEASE	500
ACTA BIOMATERIALIA	335
COLLOIDS AND SURFACES B: BIOINTERFACES	238
DRUG DELIVERY	228
ACTA PHARMACEUTICA SINICA B	221
SCIENTIFIC REPORTS	221
EXPERIMENTAL EYE RESEARCH	218
AAPS PHARMSCITECH	176
JOURNAL OF DRUG DELIVERY SCIENCE AND TECHNOLOGY	176

Tables 9 and 10 provide an overview of the most recent articles in these leading journals and their most-cited articles. A review of the recent publications in the most prominent journal reveals studies on innovative nanomaterials, such as brinzolamide nanosponges (BNS) incorporated into poloxamer 407 in situ gel (BNS-ISG), which significantly reduce intraocular pressure over extended periods.^100^ Another study examined gold nanoparticles of varying morphologies (nanorods and nanospheres) coated with polylactic-*co*-glycolic acid (PLGA) and loaded with a carbonic anhydrase inhibitor (CAI), showing that spherical nanogold particles exhibited more significant intraocular pressure reduction than commercially available eye drops.^101^ Research also included toxicity assessments of new materials like poly(3-hydroxybutyrate-*co*-3-hydroxyvalerate) nanoparticles (PHBV) loaded with acetazolamide (AECZ), another CAI, which demonstrated no functional loss in retinal cells, suggesting their suitability for controlled CAI release.^102^

**Table 9:** Latest Publications from the Most Relevant Journals

n	Source	Year	Title	ref.
1	INTERNATIONAL JOURNAL OF PHARMACEUTICS	2024	Fabrication of architectonic nanosponges for intraocular delivery of Brinzolamide: An insight into QbD driven optimization, in vitro characterization, and pharmacodynamics	(100)
		2023	Morphologic design of nanogold carriers for a carbonic anhydrase inhibitor: Effect on ocular retention and intraocular pressure	(101)
		2023	Ocular pharmacokinetics and toxicity of nanoparticular acetazolamide: In vivo distribution and safety of PHBV-ACZ nanoparticle	(102)
2	JOURNAL OF CONTROLLED RELEASE	2024	Extracellular vesicles in degenerative retinal diseases: A new therapeutic paradigm	(105)
		2024	Next generation therapeutics for retinal neurodegenerative diseases	(106)
		2023	BDNF gene delivery to the retina by cell adhesion peptide-conjugated gemini nanoplexes in vivo	(107)
3	ACTA BIOMATERIALIA	2022	Iontophoretic ocular delivery of latanoprost-loaded nanoparticles via skin-attached electrodes	(80)
		2021	Nanomedicines for the treatment of glaucoma: Current status and future perspectives	(108)
		2021	Targeted delivery of LM22A–4 by cubosomes protects retinal ganglion cells in an experimental glaucoma model	(104)
4	COLLOIDS AND SURFACES B: BIOINTERFACES	2021	Microinteraction of mucin tear film interface with particles: The inconsistency of pharmacodynamics and precorneal retention of ion-exchange, functionalized, Mt-embedded nano- and microparticles	(85)
		2021	Controlled bimatoprost release from graphene oxide laden contact lenses: < i > In vitro < /i > and in vivo studies	(95)
		2017	pH triggered controlled drug delivery from contact lenses: Addressing the challenges of drug leaching during sterilization and storage	(109)
5	DRUG DELIVERY	2023	Physicochemical properties and microinteraction between micronanoparticles and anterior corneal multilayer biological interface film for improving drug delivery efficacy: the transformation of tear film turnover mode	(83)
		2022	Sustained release of brimonidine from BRI@SR@TPU implant for treatment of glaucoma	(71)
		2022	Hyaluronic acid-enriched bilosomes: an approach to enhance ocular delivery of agomelatine via D-optimal design: formulation, in vitro characterization, and in vivo pharmacodynamic evaluation in rabbits	(110)
6	ACTA PHARMACEUTICA SINICA B	2017	Nanotechnology-based strategies for treatment of ocular disease	(111)
7	SCIENTIFIC REPORTS	2018	Topical Curcumin Nanocarriers are Neuroprotective in Eye Disease	(112)
		2018	Improving Stem Cell Delivery to the Trabecular Meshwork Using Magnetic Nanoparticles	(113)
		2018	Insights into gold nanoparticles as a mucoadhesive system	(114)
8	EXPERIMENTAL EYE RESEARCH	2023	Improved magnetic delivery of cells to the trabecular meshwork in mice	(115)
		2022	Exosomes, extracellular vesicles and the eye	(116)
		2022	Topical ocular drug delivery systems: Innovations for an unmet need	(117)
9	AAPS PHARMSCITECH	2017	PLGA Nanoparticles as Subconjunctival Injection for Management of Glaucoma	(118)
		2017	Ocular Cubosome Drug Delivery System for Timolol Maleate: Preparation, Characterization, Cytotoxicity, Ex Vivo, and In Vivo Evaluation	(103)
		2017	Development of Timolol-Loaded Galactosylated Chitosan Nanoparticles and Evaluation of Their Potential for Ocular Drug Delivery	(119)
10	JOURNAL OF DRUG DELIVERY SCIENCE AND TECHNOLOGY	2024	Advances in drug-loaded contact lenses for glaucoma: Materials, evaluation parameters, and novel drug delivery strategies with modified nanoparticles	(120)
		2023	Electrosprayed core–shell nanoparticles for sustained release fixed combination monotherapy in glaucoma treatment	(121)
		2023	Novel cross-linked nanoparticles of chitosan oligosaccharide and dextran sulfate for ocular administration of dorzolamide against glaucoma	(122)

**Table 10:** Most Cited Publications of Each Magazine among the Top 10 Most Cited

n	Source	Year	Title	No. of citations	ref.
1	INTERNATIONAL JOURNAL OF PHARMACEUTICS	2015	Cationic solid lipid nanoparticles enhance ocular hypotensive effect of melatonin in rabbit	72	(123)
2	JOURNAL OF CONTROLLED RELEASE	2016	In vitro and in vivo evaluation of novel implantation technology in hydrogel contact lenses for controlled drug delivery	149	(124)
3	ACTA BIOMATERIALIA	2019	Effect of gold nanoparticles on timolol uptake and its release kinetics from contact lenses: In vitro and in vivo evaluation	81	(96)
4	COLLOIDS AND SURFACES B: BIOINTERFACES	2014	Physicochemical characterization of epigallocatechin gallate lipid nanoparticles (EGCG-LNs) for ocular instillation	86	(125)
5	DRUG DELIVERY	2019	Proniosomal gel-derived niosomes: an approach to sustain and improve the ocular delivery of brimonidine tartrate; formulation, in vitro characterization, and in vivo pharmacodynamic study	64	(126)
6	SCIENTIFIC REPORTS	2018	Topical Curcumin Nanocarriers are Neuroprotective in Eye Disease	69	(112)
7	ACTA PHARMACEUTICA SINICA B	2017	Nanotechnology-based strategies for treatment of ocular disease	221	(127)
8	EXPERIMENTAL EYE RESEARCH	2019	Thermosensitive chitosan-gelatin-based hydrogel containing curcumin-loaded nanoparticles and latanoprost as a dual-drug delivery system for glaucoma treatment	82	(128)
9	JOURNAL OF DRUG DELIVERY SCIENCE AND TECHNOLOGY	2020	In situ gel containing Bimatoprost solid lipid nanoparticles for ocular delivery: in vitro and ex-vivo evaluation	42	(129)
10	AAPS PHARMSCITECH	2017	Ocular Cubosome Drug Delivery System for Timolol Maleate: Preparation, Characterization, Cytotoxicity, Ex Vivo, and In Vivo Evaluation	75	(103)

Additional studies focus on drug delivery systems, such as cubesomes for timolol administration^103^ and targeted delivery of LM22A-4 to protect retinal cells in degenerative diseases.^104^ Research on ocular iontophoresis is also progressing, using mild electrical currents to increase ocular permeability and enhance the absorption of therapeutic agents like latanoprost.^80^ These publications underscore this field’s dynamic and evolving nature, driven by the need for more effective and less invasive glaucoma treatments.

#### 3.1.4 Documents

The bibliometric analysis of 324 documents related to micro- and nanotechnology applications in glaucoma treatment highlights a growing and diverse field. Covering publications from 2014 to 2024, the study shows an average publication year of 2019, indicating that this area has gained significant attention in the past decade. The diversity of the 147 journals publishing these works reflects the interdisciplinary nature of the topic. Academic impact varies widely across these documents (Table 11), with the most cited paper being a comprehensive review of nanotechnology-based strategies for eye diseases.^127^ The second most cited is experimental research investigating contact lens-based drug delivery systems,^124^ a promising alternative for glaucoma treatment.^33^

**Table 11:** Most Cited Documents

Title	Year	Authors	Journal	Citations	Cluster	ref.
Nanotechnology-based strategies for treatment of ocular disease	2017	Weng, Yuhua and Liu, Juan and Jin, Shubin and Guo, Weisheng and Liang, Xingjie and Hua, Zhongbo	ACTA PHARMACEUTICA SINICA B	221	2	(127)
In vitro and in vivo evaluation of novel implantation technology in hydrogel contact lenses for controlled drug delivery	2016	Maulvi, Furqan A. and Lakdawala, Dhara H. and Shaikh, Anjum A. and Desai, Ankita R. and Choksi, Harsh H. and Vaidya, Rutvi J. and Ranch, Ketan M. and Koli, Akshay R. and Vyas, Bhavin A. and Shah, Dinesh O.	JOURNAL OF CONTROLLED RELEASE	149	4	(124)
Drug delivery to the eye: what benefits do nanocarriers offer?	2017	Joseph, Rini Rachel and Venkatraman, Subbu S.	NANOMEDICINE	124	5	(130)
Nanotherapies for the treatment of ocular diseases	2015	Reimondez-Troitino, S. and Csaba, N. and Alonso, M. J. and de la Fuente, M.	EUROPEAN JOURNAL OF PHARMACEUTICS AND BIOPHARMACEUTICS	121	5	(131)
Sustained drug release by contact lenses for glaucoma treatment-A review	2015	Carvalho, I. M. and Marques, C. S. and Oliveira, R. S. and Coelho, P. B. and Costa, P. C. and Ferreira, D. C.	JOURNAL OF CONTROLLED RELEASE	112	4	(132)
Glaucoma: Current treatment and impact of advanced drug delivery systems	2019	Yadav, Khushwant S. and Rajpurohit, Rahul and Sharma, Sushmita	LIFE SCIENCES	111	5	(133)
Application of lipid nanoparticles to ocular drug delivery	2016	Battaglia, Luigi and Serpe, Loredana and Foglietta, Federica and Muntoni, Elisabetta and Gallarate, Marina and Del Pozo Rodriguez, Ana and Angeles Solinis, Maria	EXPERT OPINION ON DRUG DELIVERY	100	5	(134)
Curcumin: Therapeutical Potential in Ophthalmology	2014	Pescosolido, Nicola and Giannotti, Rossella and Plateroti, Andrea Maria and Pascarella, Antonia and Nebbioso, Marcella	PLANTA MEDICA	95	2	(135)
Dually functional hollow ceria nanoparticle platform for intraocular drug delivery: A push beyond the limits of static and dynamic ocular barriers toward glaucoma therapy	2020	Luo, Li-Jyuan and Duc Dung Nguyen and Lai, Jui-Yang	BIOMATERIALS	86	5	(76)
Physicochemical characterization of epigallocatechin gallate lipid nanoparticles (EGCG-LNs) for ocular instillation	2014	Fangueiro, Joana F. and Andreani, Tatiana and Fernandes, Lisete and Garcia, Maria L. and Egea, Maria A. and Silva, Amelia M. and Souto, Eliana B.	COLLOIDS AND SURFACES B-BIOINTERFACES	86	3	(125)

**Figure 10:**
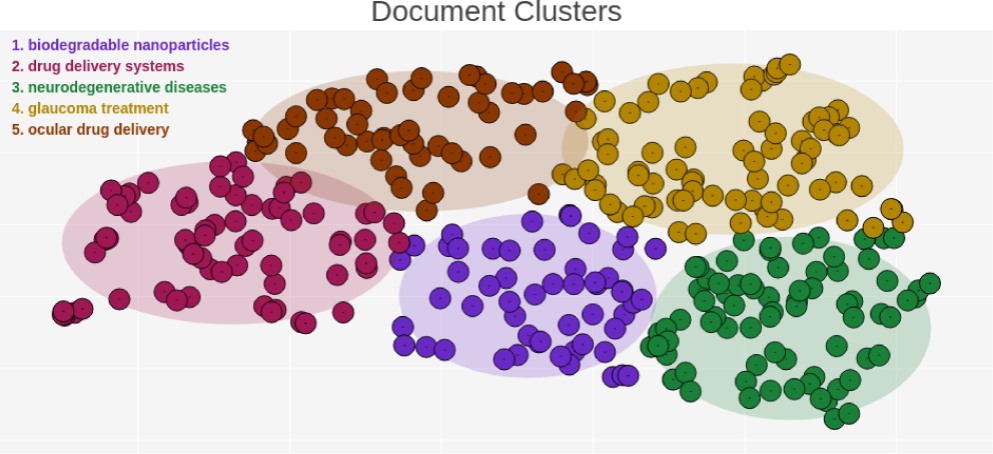
Cluster of documents divided by subtheme.

Figure 10 illustrates a clear division of documents into five main thematic clusters: (1) biodegradable nanoparticles, (2) drug delivery systems, (3) neurodegenerative diseases, (4) glaucoma treatment, and (5) ocular drug delivery. The overlap between clusters, such as drug delivery systems and ocular drug delivery, reflects the interconnected nature of these themes.

#### 3.1.5 Thematic Trends

**Figure 11:**
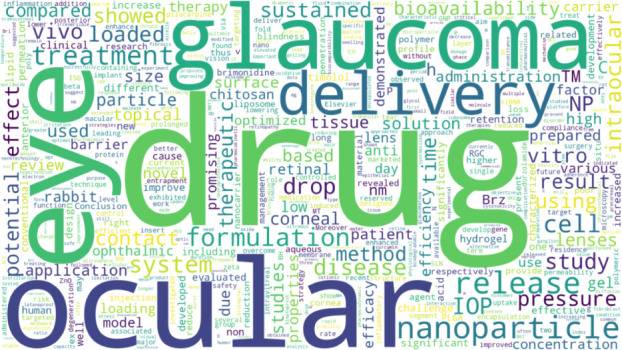
Word cloud of the most frequent keywords in abstracts.

Keyword analysis is crucial for identifying research trends within this topic. Table 12 ranks keywords based on their frequency in the abstracts of the bibliometric study, with terms such as “drug,” “ocular,” “eye,” “glaucoma,” and “delivery” most closely aligned with the topic (Figure 11).

**Table 12:** Order of Importance of the Keywords Contained in the Abstracts

Rank	Words	Importance
1	drug	1
2	ocular	0.5893037336024218
3	eye	0.5428859737638748
4	glaucoma	0.5105953582240161
5	delivery	0.4601412714429869
6	nanoparticle	0.425832492431887
7	release	0.3975782038345106
8	treatment	0.30171543895055497
9	formulation	0.2674066599394551
10	cell	0.2532795156407669
11	system	0.23612512613521694
12	IOP	0.23208879919273462
13	drop	0.218970736629667
14	disease	0.21796165489404642
15	study	0.2058526740665994
16	loaded	0.20080726538849647
17	vivo	0.19979818365287588
18	intraocular	0.1987891019172553
19	NP	0.19576185671039353
20	using	0.18869828456104945
21	sustained	0.18869828456104945
22	pressure	0.18668012108980828
23	showed	0.17961654894046417
24	effect	0.17658930373360243
25	vitro	0.17558022199798184

**Figure 12:**
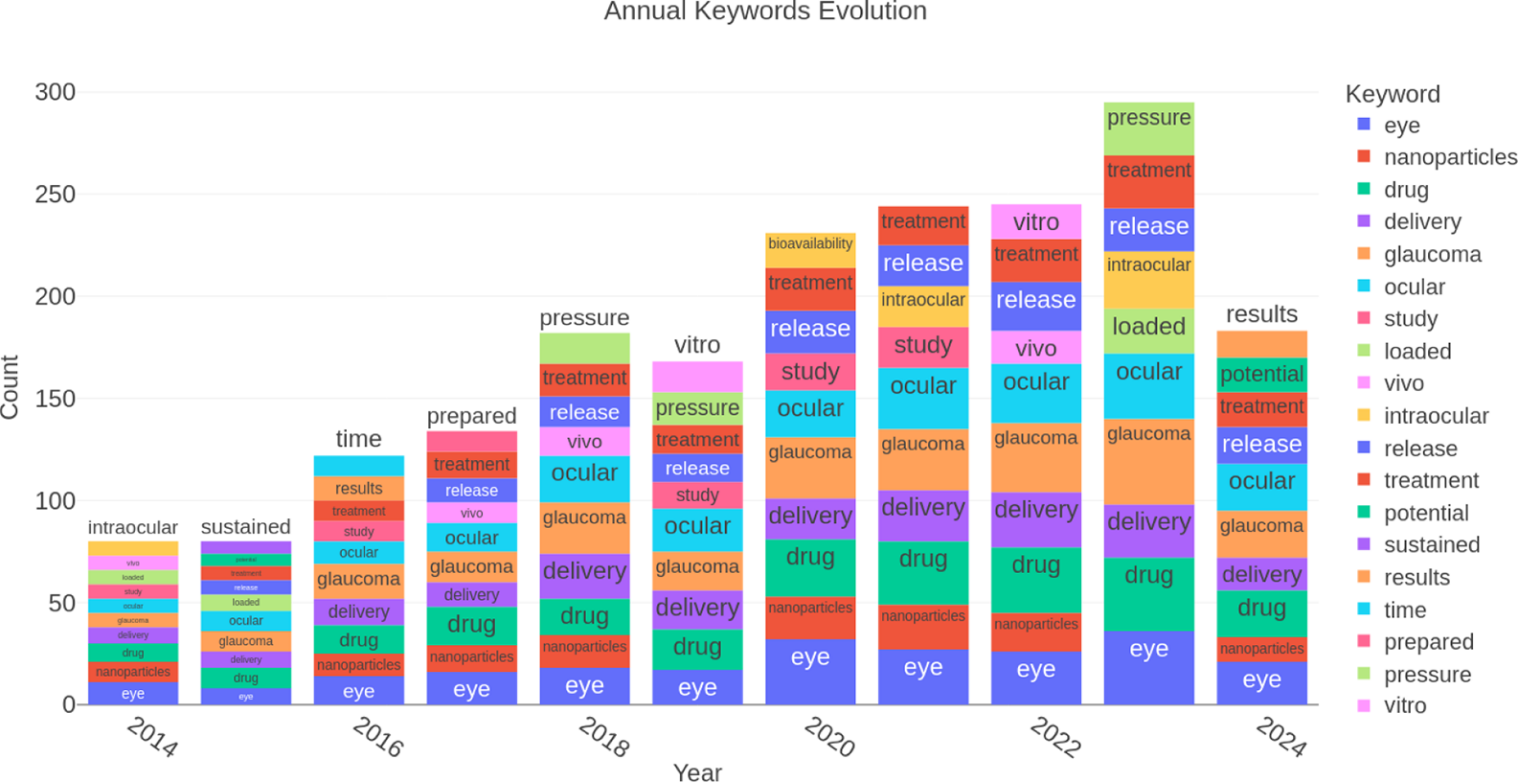
Yearly evolution of keywords.

**Figure 13:**
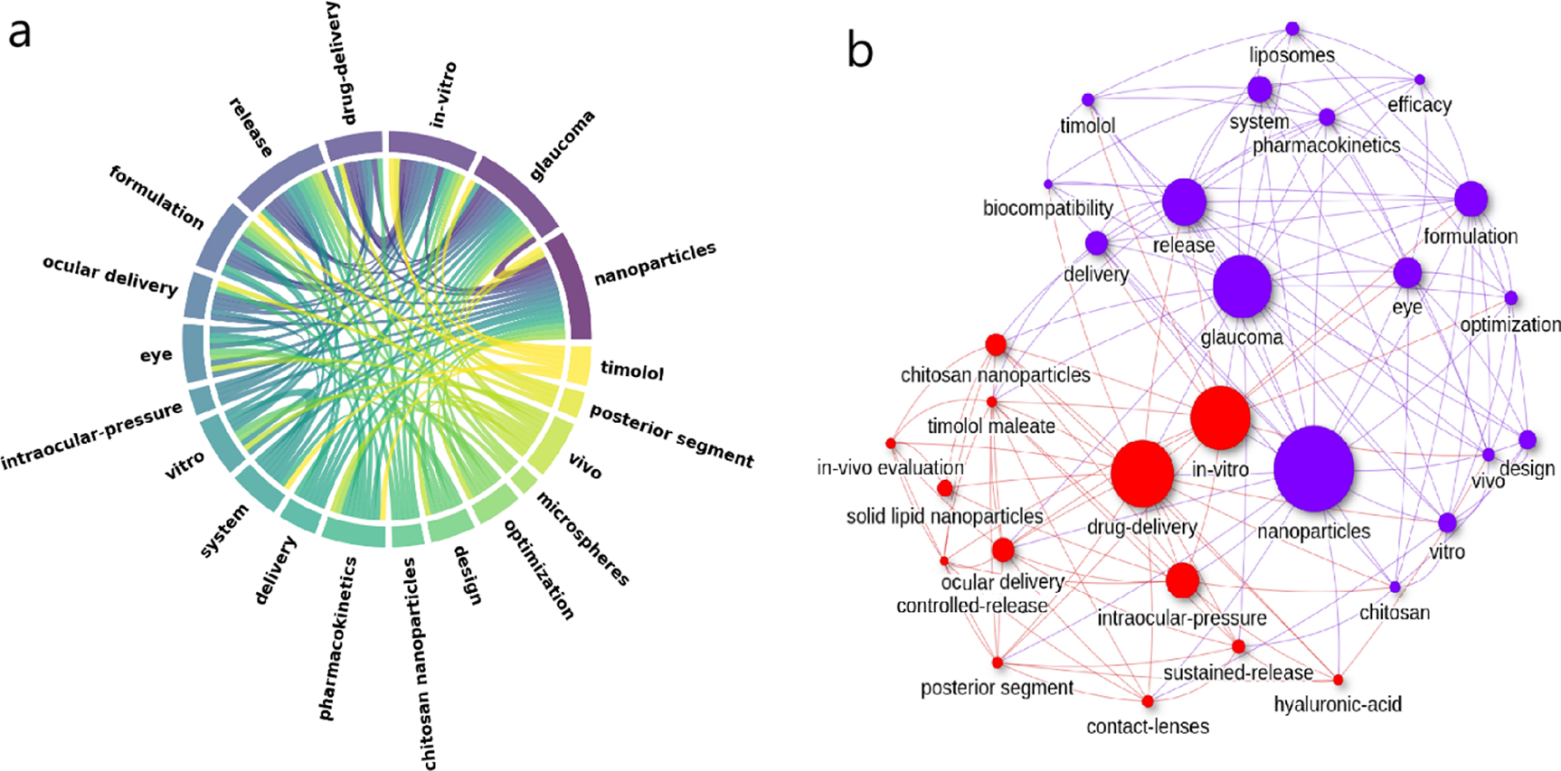
Keyword analysis. (a) Chord diagram illustrating relationships between keywords; (b) neural network displaying keyword co-occurrence patterns.

Looking at the year-to-year trends in keywords, Figure 12 shows that terms such as “ocular,” “glaucoma,” “treatment,” and “release” remained prevalent throughout the study period. However, starting in 2020, there was a noticeable increase in terms such as “bioavailability” and “nanoparticles.” Figures 13a,b illustrates the relationships between keywords and reveal a recent focus on nanoparticles and targeted delivery systems. The increase in the number of clinical trials suggests that these technologies are approaching broader clinical applications, potentially transforming available treatment options.

**Figure 14:**
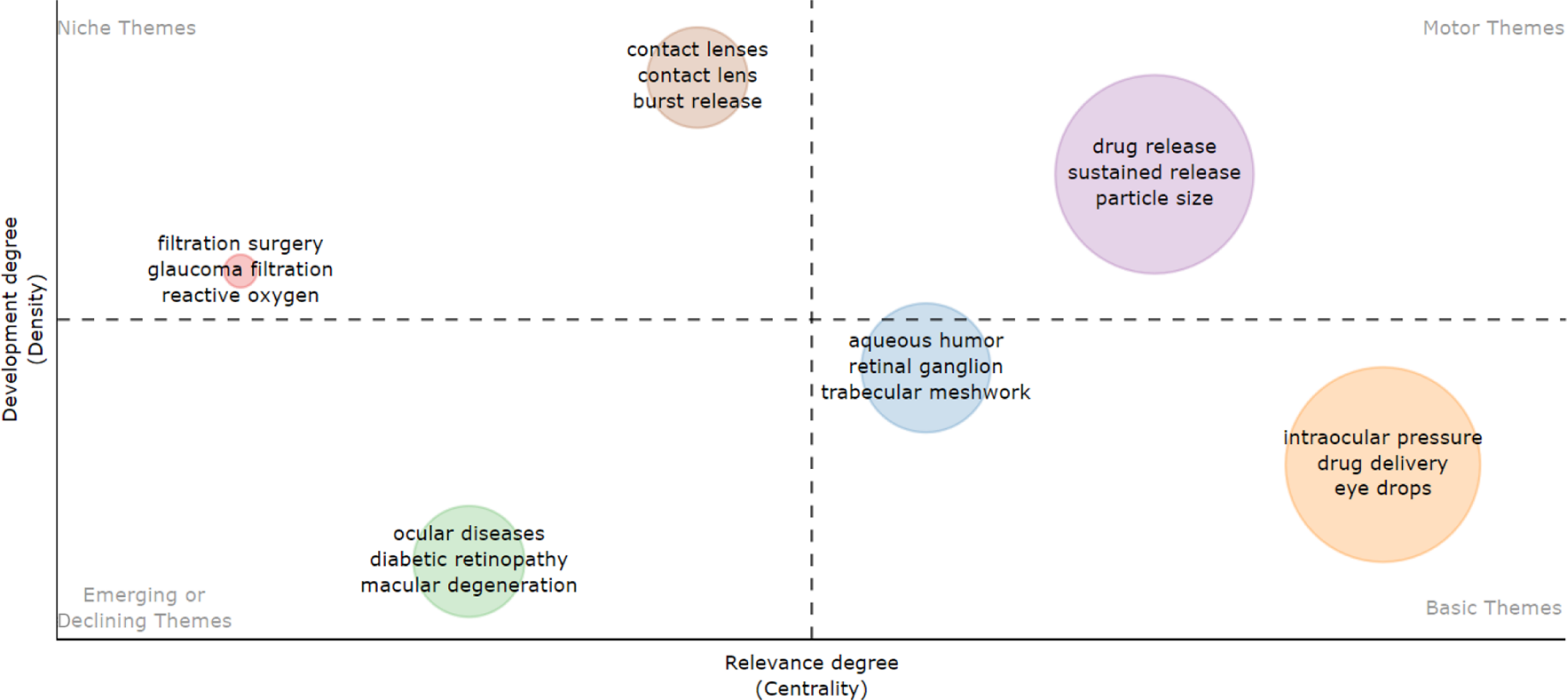
Thematic map is divided into quadrants.

Figure 14 presents a thematic map that categorizes keywords into four quadrants based on their centrality and level of development. In the Basic Themes quadrant, terms such as “intraocular pressure,” “drug delivery,” and “eye drops” are well-established, central concepts that form a solid foundation in ocular disease treatment research, highlighting their ongoing relevance and development. In the Emerging or Declining Themes quadrant, terms such as “ocular diseases,” “diabetic retinopathy,” and “macular degeneration” suggest a potential shift in research focus, as emerging technologies may be drawing attention to other therapeutic approaches or more innovative methodologies. The Motor Themes quadrant features keywords such as “drug release,” “sustained release,” and “particle size,” which lie at the heart of current innovations and indicate a growing emphasis on improving controlled drug release, essential for advancements in nanotechnology-based eye disease treatments. Lastly, the Niche Themes quadrant includes “contact lenses” and “burst release.” Although less central, these areas continue to grow in specific fields, representing smaller research domains with significant potential for future expansion.

### 3.2 Microtechnology Overview

This section offers an overview of microtechnology applications in glaucoma treatment, as identified through the bibliometric analysis discussed previously. It explores devices such as microemulsions, microneedles, and mucoadhesive microparticles. The subtopics are organized by microdevice type and their respective applications, reflecting the relatively low number of documents related to microtechnology obtained in the bibliometric study.

#### 3.2.1 Microparticles for Controlled Release

The use of microparticles (MPs) for controlled drug release is a promising approach to glaucoma treatment. One study highlights the development of PLGA microparticles loaded with latanoprost and dexamethasone, created via capillary microfluidics. These particles, with an average diameter of 150 μm, provided controlled release for up to 5 days and extended ocular surface retention due to a chitosan coating.^136^

Other studies have explored different methodologies, such as mucoadhesive microparticles,^137,138^ which show strong potential for drug retention on the ocular surface.^137^ In one application, PLGA and polyethylene glycol (PEG) MPs were used for the topical delivery of dorzolamide, a CAI. This study compared the efficacy of these MPs to a commercial dorzolamide eye drop formulation. Results showed that the MPs achieved a maximum IOP reduction of 35% and doubled the reduction duration compared to commercial eye drops.^138^

Another study evaluated the biocompatibility and safety of PLGA or poly(lactic acid) (PLA) microparticles administered via intravitreal injection in New Zealand rabbits. The study examined the effects of these microparticles at different doses (low, medium, and high), including variations loaded with erythropoietin. Evaluations included clinical examinations, retinal histology, and electroretinography, revealing that the microparticles did not significantly alter retinal structure or function, demonstrating their safety.^139^

#### 3.2.2 Microspheres and Their Application in Glaucoma

A recent study explored the use of microspheres composed of rapamycin (RAPA), chitosan (CS), and calcium alginate (CA) to prevent scarring after glaucoma filtering surgery in rabbits. Results showed that these microspheres effectively reduced fibroblast formation and collagen fiber proliferation. They also demonstrated a cumulative release rate of 94.07% over 49 days, maintaining low IOP levels for an extended period while preserving epithelial integrity.^140^

Another approach investigated the use of microspheres for controlled release utilizing montmorillonite-encapsulated betaxolol (Mt-BH) and Eudragit microspheres. This formulation achieved a prolonged release of up to 12 h, with lower toxicity and more effective IOP reduction.^141^ Additionally, the safety of polymeric nanoparticles, such as PLGA/PLA microspheres, was evaluated by using intravitreal injections in rabbits. Results confirmed their biocompatibility and safety, as these microspheres did not cause histological changes or functional damage to the retina.^142^

#### 3.2.3 Microemulsions for Ocular Drug Delivery

Beyond microparticles and microspheres, microemulsions have shown promising potential for glaucoma treatment. Recent studies demonstrate various applications of microemulsions, such as using them to soak contact lenses for travoprost delivery. These lenses provided sustained release for up to 120 h, compared to only 48 h with conventional soaking methods, and maintained key physical properties, including optical transparency.^143^ Another study used microemulsions to soak contact lenses for timolol delivery, achieving prolonged IOP reduction lasting up to 96 h.^144^

In additional research, brimonidine microemulsions were developed to transition from a water-in-oil structure to liquid crystals and, finally, to oil-in-water emulsions when diluted in the eye. This study showed that these microemulsions enhance corneal permeability and increase precorneal retention time.^145^

#### 3.2.4 Microneedles for Drug Delivery

Finally, one study focused on developing an eye patch with microneedles to deliver pilocarpine through the corneal membrane. The microneedles were arranged in a pyramidal array of 25 needles, significantly enhancing pilocarpine permeability compared with the solution formulation. Ex vivo studies on pig eyeballs demonstrated higher drug retention in the aqueous humor after applying the eye patch.^146^

### 3.3 Overview of Nanotechnology

This section examines recent advances and applications of nanodevices in glaucoma treatment, building on the bibliometric analysis discussed earlier and focusing on innovative architectures such as nanovesicles, nanocomposites, and various drugs and nanoparticle types.

#### 3.3.1 Innovative Nanodevices for Eye Therapy

In recent years, nanotechnology has shown great promise in enhancing the therapeutic efficacy of ocular drugs, particularly for conditions affecting both the anterior and posterior eye segments. In this context, nanomedicine and nanodrugs have gradually emerged, offering unmatched advantages, including the ability to cross ophthalmic barriers, reach targeted tissues, and release therapeutic agents precisely.^108^ Recent approaches feature innovative nanostructured architectures, such as nanovesicles, nanosponges, and nanorods.

One study focused on ultra-deformable nanovesicles to improve ocular drug delivery, specifically developing and evaluating an ocular formulation of Nebivolol HCl (NBV)—a selective beta-1 blocker with vasodilatory properties.^147^ Encapsulated in plastic nanovesicles (SNVs) and incorporated into a gel, NBV was used as an anti-inflammatory agent to treat glaucoma complications, showing notable improvements in ocular permeability, absorption, anti-inflammatory activity, and reduced intraocular pressure.^148^ Additionally, the development of porous nanocomposites (nanosponges) loaded with brinzolamide and incorporated into poloxamer 407 gels was explored to overcome anatomical barriers of the eye. Ex vivo and in vivo studies in animal models confirmed the formulation’s safety and efficacy, showing significant reductions compared to commercial models.^100^ Advanced nanomaterials are also gaining traction in ophthalmology, including gold-based contrast agents with a rod-like structure, which optimize ophthalmic imaging techniques such as optical coherence tomography and photoacoustic microscopy,^149^ and bioceramics used in orbital surgery.^150^

Another innovative solution recently studied is the development of contact lenses for controlled drug release.^72^ Some studies using latanoprost have shown effectiveness in controlled release and IOP reduction, as seen with lenses containing latanoprost composites and latanoprost-timolol, activated by lysozyme (Lyz) enzymes in tear fluid,^151^ or with lenses loaded with PEGylated solid lipid nanoparticles.^152^ Drugs such as brimonidine, a selective alpha-2 adrenergic receptor agonist that inhibits aqueous humor production and increases uveoscleral outflow,^153^ have also been incorporated into contact lenses. For example, silicone contact lenses loaded with silica nanoparticles demonstrated positive results in controlled release, safety, and efficacy compared to conventional methods.^154^ The application of drug-loaded lenses is broad, covering a range of medications. Timolol maleate, embedded in nanoparticles decorated with lauric acid and coated with albumin, achieved a prolonged release lasting up to 6 days, offering a promising alternative.^155^

From this perspective, nanotechnology holds promise due to its potential to extend drug retention time on the ocular surface, enable controlled release, improve ocular absorption of anti-inflammatory drugs, and overcome barriers like the blood-retinal barrier—all while demonstrating low toxicity in preclinical studies.^156−159^ Research in this area has led to the development of various drug delivery systems for glaucoma.

#### 3.3.2 Therapeutic Agent Delivery Systems in Glaucoma

Recent research on nanoparticle-based drug delivery systems has developed nanodevices with diverse morphologies, functionalities, and applications. One example is chitosan nanoparticles (Cs NPs) used to encapsulate hydrochloric fasudil, a selective Rho-kinase inhibitor (ROCK) and serine/threonine enzyme that relaxes the trabecular meshwork by increasing aqueous humor outflow.^160,161^ Studies have shown that Cs NPs significantly improve drug permeation through the cornea.^162^

Other studies have focused on enhancing the ocular bioavailability of timolol maleate (TM) to reduce the administration frequency. For instance, magnesium hydroxide nanoparticles have increased drug penetration through the cornea, leading to significant IOP reduction without toxic effects on the eye.^163^ Another approach combines TM with brinzolamide in a nanostructured lipid carrier (NLC) system, improving the release and permeation of both drugs and providing better IOP control.^164^ Additional studies explore using NPs with TM for glaucoma treatment, as summarized in Table 13.

**Table 13:** Overview of the Use of NPs in the Treatment of Glaucoma

Drugs	Nanodevices	Functions	Results	ref
Brinzolamide	Core–shell NPs	Sustained release	Efficacy in brinzolamide delivery, reducing IOP with controlled release	(166)
	Mucoadhesive NPs (chitosan-pectin)	Increase drug adherence to the ocular mucosa, improving retention time in the eye.	Better control over intraocular pressure due to higher retention in the eye.	(167)
	Architectural nanosponges	Improve solubility and controlled release of Brinzolamide, especially in challenging ocular environments.	Enhanced drug delivery and greater efficacy in reducing intraocular pressure over time.	(100)
	Nanoliposomes	Enhance tissue penetration for better efficiency in drug delivery.	Better efficiency in drug delivery.	(168)
	Biodegradable PEG–PSA NPs (polyethylene glycol-*co*-polysuccinate)	Improve drug and gene therapy efficiency through codelivery of Brinzolamide and miRNA-124.	Better therapeutic outcomes, addressing both pressure and neurodegenerative effects.	(169)
	Nanostructured lipid carrier	Improve ocular bioavailability, permeation, and precorneal resistance time of Timolol Maleate and Brinzolamide.	Greater efficacy compared to individual delivery.	(164)
Dorzolamide	Chitosan NPs in situ gels	Increase ocular adhesion and prolong the residence time of Dorzolamide in the eye.	The formulation showed superior corneal retention and sustained drug release.	(173)
	Chitosan oligosaccharide and dextran sulfate NPs (CSO–DS)	Improve mucoadhesive adhesion, penetration, and bioavailability of Dorzolamide.	Significant reduction of IOP (41.56%) with prolonged drug release for 12 h, also safe and suitable for ocular administration based on HET-CAM tests.	(122)
	Cationic nanoemulsions	Improve ocular delivery of Dorzolamide, optimizing the formulation using Box-Behnken design.	Demonstrated sustained release of Dorzolamide and better mucoadhesive adhesion compared to the pure solution and commercial eye drops, without causing ocular irritation in rabbit tests.	(172)
	Chitosan NPs derived from glycolic acid	Improve the pharmacokinetic and pharmacological parameters of Dorzolamide.	Optimized formulations showed more excellent drug absorption in the aqueous humor and prolonged control of IOP, superior to the commercial product Trusopt.	(171)
Brimonidine	PLGA-TGPS NPs (poly(lactic-*co*-glycolic acid) + vitamin E-TGPS)	Improve mucoadhesive properties and increase drug retention capacity.	Demonstrated sustained release of brimonidine and increased corneal permeability, with a 34.46% IOP reduction, approximately 3 times more effective than commercial eye drops.	(174)
	Nanostructured lipid carriers (NLCs)	Increase ocular resistance time and corneal penetration.	Showed greater encapsulation efficiency and stability compared to solid NPs and commercial eye drops, reducing IOP in a sustained manner.	(175)
	Lipid-DNA NPs	Improve surface adhesion and provide controlled release of brimonidine.	Significantly increased corneal affinity and brimonidine release, resulting in higher IOP reduction efficacy without ocular toxicity.	(176)
	Silica NPs incorporated into contact lenses	Provide controlled release of brimonidine without altering the optical properties of the lens.	Demonstrated controlled release for up to 144 h with high brimonidine concentrations maintained for 96 h in a rabbit tear fluid model, with no detected toxicity.	(154)
Dexamethasone	Chitosan NPs	Improve the ocular bioavailability of dexamethasone sodium.	Presented sustained dexamethasone release, with approximately 55.73% of the drug released over 22 days. The release profile was suitable for therapeutic use in anti-inflammatory conditions.	(177)
	PLGA NPs (poly(lactic-*co*-glycolic acid))	Co-release dexamethasone and melatonin using coaxial electrospray method.	Resulted in sustained release of dexamethasone and melatonin, without initial burst release, and increased retinal penetration. Significantly reduced IOP, showing neuroprotective potential.	(178)
	Dexamethasone NPs	Use as an alternative to mitomycin C (MMC) post-trabeculectomy to control inflammation and postoperative fibrosis.	Provided effective IOP reduction after surgery, with no significant differences in surgical success rates.	(179)
	Biodegradable dexamethasone sodium phosphate NPs (DSP-Zn-NP)	Controlled release of dexamethasone with dense polyethylene glycol (PEG) coating.	A single subconjunctival administration prevented suture-induced corneal neovascularization in rats for 2 weeks.	(180)
Bimatoprost	Gold NPs (GNPs)	Load bimatoprost into contact lenses, improve retention, and reduce abrupt release.	The loaded lenses showed sustained release of bimatoprost for up to 72 h and better retention in tear fluid compared to conventional methods.	(181)
	Solid lipid NPs (SLNs)	Load bimatoprost into lipid NPs incorporated in situ gel, improving ocular release and IOP control.	The formulation showed prolonged, nonirritant release with potential for better glaucoma management.	(129)
	Nanovesicles (BMT-NV)	Release bimatoprost via thermosensitive subconjunctival gel implants.	Demonstrated IOP reduction for up to 2 months after a single injection, with no signs of ocular irritation.	(182)
	Liposomes and SLNs	Compare liposomes and SLNs encapsulated in thermosensitive in situ gel for sustained bimatoprost release.	Liposomes showed greater encapsulation efficiency and better release control without initial burst, with IOP reduction for up to 18 h.	(89)
Latanoprost	PLGA NPs	Load latanoprost into PLGA NPs and increase ocular penetration.	Demonstrated prolonged IOP reduction for more than 7 days with a single administration, with 23 times greater efficacy than commercial eye drops.	(80)
	Curcumin NPs (CUR-NPs) in thermosensitive hydrogels	Reduce oxidative stress in glaucoma treatment through the release of latanoprost and curcumin.	Showed sustained release, reduced oxidative stress in trabecular meshwork cells, and in vivo biocompatibility.	(128)
	PEGylated solid lipid NPs (PEG-SLNs)	Improve latanoprost loading into contact lenses and sustain drug release.	Showed prolonged drug release for up to 96 h and higher ocular concentration than conventional eye drops.	(152)
Travoprost	Liquid nanocrystals (LCNs)	Improve ocular permeability and bioactivity of travoprost.	Reduced IOP for up to 72 h with greater bioavailability than commercial Travatan eye drops.	(183)
	Nanoemulsions	Improve bioavailability and controlled release of travoprost.	Showed more excellent ocular absorption and prolonged IOP reduction without irritation.	(184)
	Self-organized DNA NPs	Prolong surface adhesion and increase the drug delivery rate.	Travoprost delivery was two times higher compared to the pure drug, with proven biosafety.	(185)
Betaxolol	Multilayer NPs (RAMt-BH-HA/CS@ED)	Sustained release of Betaxolol Hydrochloride (BH) with high encapsulation efficiency via refined acid-treated montmorillonite (RAMt), hyaluronic acid/chitosan NPs (HA/CS), and Eudragit (ED) films.	Prolonged IOP reduction for over 12 h, with excellent ocular retention properties and proven safety in animal models.	(87)
	Multifunctional NPs (MMt-BH-HA/CS-ED)	Increase the bioavailability of BH on the ocular surface through two-stage release.	Prolonged release and lower precorneal diffusion rate, increased drug retention time and IOP reduction efficacy.	(186)
Puerarin	Human albumin NPs (PUR-HSA-NPs)	Improve ocular drug release with thermoresponsive gel loading.	Showed 80.7% encapsulation efficiency, and the system resulted in continuous IOP reduction over a long period. The NP system also reduced cell apoptosis, minimizing cell death.	(187)
Pilocarpine	Zinc oxide/PVP NPs (ZnO/PVP)	Sustained release of pilocarpine hydrochloride (PHCl) through collagen shields with NPs.	Controlled PHCl release for 14 days with good biocompatibility and no ocular toxicity.	(188)

Brinzolamide (Brz), a carbonic anhydrase inhibitor, reduces aqueous humor formation in the eye.^165^ Many studies have focused on nanoparticulate delivery systems for Brz, including core–shell nanoparticles composed of phosphatidylserine and polylactic-*co*-glycolic acid (PLGA),^166^ chitosan-pectin mucoadhesive nanocapsules,^167^ nanoliposomes encapsulating a brinzolamide-hydroxypropyl-β-cyclodextrin inclusion complex,^168^ and biodegradable NPs for codelivery of Brz and miRNA-124.^169^

Another carbonic anhydrase inhibitor, dorzolamide,^170^ has been explored in various nanotechnology-based studies. Research has focused on developing self-organizing nanostructures of dorzolamide and l-α-phosphatidylcholine,^171^ cationic nanoemulsions,^172^ isolated chitosan NPs,^173^ and formulations combining chitosan with dextran sulfate,^122^ aiming to improve pharmacokinetic parameters, extend pharmacological action,^171^ and enhance adhesion and penetration into ocular tissues.^122^ These formulations demonstrated significant IOP reduction, increased adhesion to ocular mucus, and improved ocular permeability and penetration.^166−169^

Besides beta-blockers such as timolol maleate and enzyme inhibitors (e.g., rho-kinase and carbonic anhydrase inhibitors), agonists are also crucial in glaucoma treatment. Brimonidine, a well-studied alpha-2 adrenergic agonist, has been investigated with various nanotechnological devices. Studies include brimonidine-loaded PLGA-TPGS nanoparticles in thermosensitive in situ gels,^174^ nanostructured lipid carriers,^175^ and functionalized lipid-DNA nanoparticles.^176^

Other drugs, such as corticosteroids and prostaglandin analogues, have also been investigated in glaucoma treatment studies utilizing nanodevice methodologies. Table 13 provides additional details about the use of these drugs. Their selection and ordering in the table are based on the number of articles covering each drug, with priority given to those with the most studies, as identified through the bibliometric analysis.

## 4 Strengths and Limitations

Over the years, emerging nanorobotic applications in ophthalmic therapy have shown strengths and limitations. Strengths include advances in nanotechnology that revolutionize drug delivery systems and precise research approaches. Limitations, however, present challenges in several areas, including biocompatibility testing, the safety of emerging technologies, manufacturing and scalability issues, and limited clinical data.

Most anterior segment eye diseases are treated with eye drops. However, eye drops are often associated with low ocular bioavailability due to their short residence time on the ocular surface. Therapeutic contact lenses for ocular drug administration have gained considerable attention as a versatile, cost-effective, and biocompatible option. Therapeutic contact lenses can improve the ocular bioavailability of medications by maintaining close contact with the cornea.^93^

One innovative approach involves using NH2-MIL(Fe)-88, a metal–organic framework (MOF), as a delivery carrier for brimonidine. NH2-MIL(Fe)-88 particles possess mucoadhesive properties, enabling prolonged retention in the preocular space when administered topically. These particles can encapsulate drugs within their micropores, facilitating a sustained drug release. In vivo testing in rabbit eyes demonstrated that NH2-MIL(Fe)-88 particles loaded with brimonidine significantly improved ocular bioavailability compared to Alphagan P, a commercially available brimonidine eye drop.^72^

Another strength in this field is the efficacy of microneedle eye patches (MOPs) in delivering pilocarpine HCl through the corneal membrane. MOPs are designed to resemble commercial contact lenses. Ex vivo studies on porcine eyeballs showed that pilocarpine availability in the aqueous humor within 30 min of MOP application was significantly greater (249 ± 85 μg/mL) than that achieved with the solution formulation (46 ± 9 μg/mL; *p* < 0.05). These findings suggest that MOPs hold potential as an ophthalmic drug delivery system.^124^

Another innovative approach is the development of nano eye drops for managing glaucoma progression. This ophthalmic nanoformulation uses hollow ceria nanoparticles, dual-functionalized with chitosan and ZM241385, and subsequently loaded with pilocarpine for controlled release. Compared to commercial eye drops, these nano eye drops, when applied topically, demonstrate a significant improvement in slowing experimental glaucoma progression.^114^

While trabecular meshwork (TM) cell therapy shows promise for controlling IOP, current cell administration techniques face limitations due to low delivery efficiency. To address this, a magnetic delivery technique has been developed to reduce unwanted off-target cell delivery effects. Mesenchymal stem cells (MSCs), labeled with superparamagnetic iron oxide nanoparticles (SPIONs), were injected into the anterior chamber and then magnetically directed to the TM using a focused “dot magnet” apparatus.^93^

However, this method has limitations. The study did not report essential injection parameters, such as flow rate or details of image processing and quantification, including precise criteria for defining the “anterior chamber angle” and its limits, making a direct comparison with previous results difficult. In addition, when quantifying delivery specificity, the researchers used sagittal images from which lenses were removed, thereby excluding potential off-target delivery to the lens from specificity calculations, another limitation noted in recent studies.^93^

Topical administration via the conjunctival cul-de-sac is the most common route for ocular drug delivery. Despite its accessibility, the eye is highly protected from foreign substances, including therapeutic agents, by effective mechanisms such as blinking, induced tearing, tear turnover, and nasolacrimal drainage. These defenses rapidly clear substances from the eye’s surface. The cornea also serves as a robust barrier, limiting the ocular bioavailability of many drugs and underscoring the need for innovative drug delivery systems (DDS), particularly nanotechnology-based strategies.^101^

While eye drops are widely used to deliver drugs to the anterior segment of the eye, they often suffer from low bioavailability due to their short contact time and rapid clearance by tears. Effective drug delivery to the posterior segment of the eye presents additional challenges. It often requires alternative routes such as periocular and intravitreal administration, with the blood-retinal barrier being a significant barrier to systemic drug delivery.^112^

These observations highlight the progress and results achieved through research and studies of emerging nanorobotic applications in ophthalmic therapy. The contributions of these studies are substantial and offer the potential for future innovation despite certain limitations. These limitations can serve as both cautionary insights and areas for improvement, ultimately helping refine and enhance these methods.

## 5 Patents

### 5.1 Nanotech

**Figure 15:**
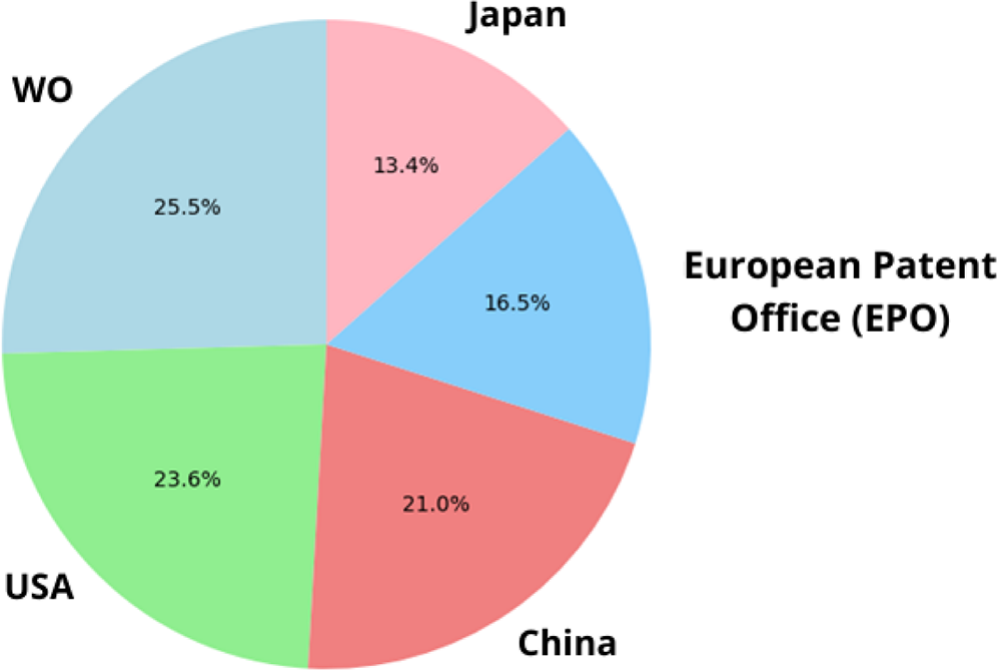
Percentage distribution of patent registrations by country of application in nanotechnology.

**Figure 16:**
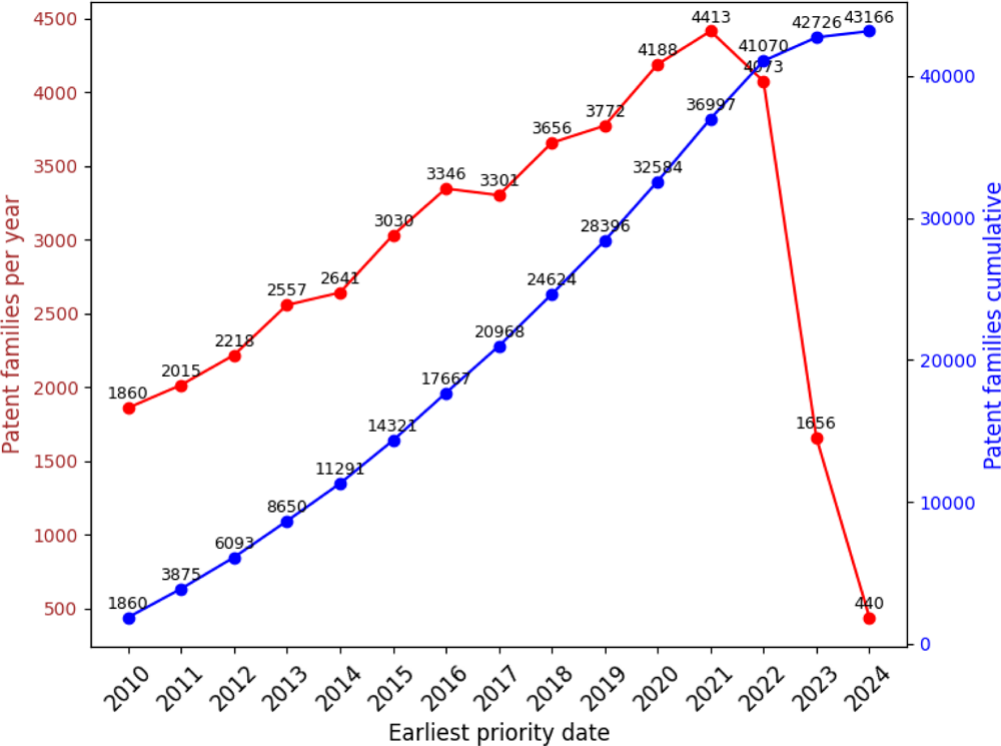
Annual and cumulative numbers of nanotechnology patent families.

**Figure 17:**
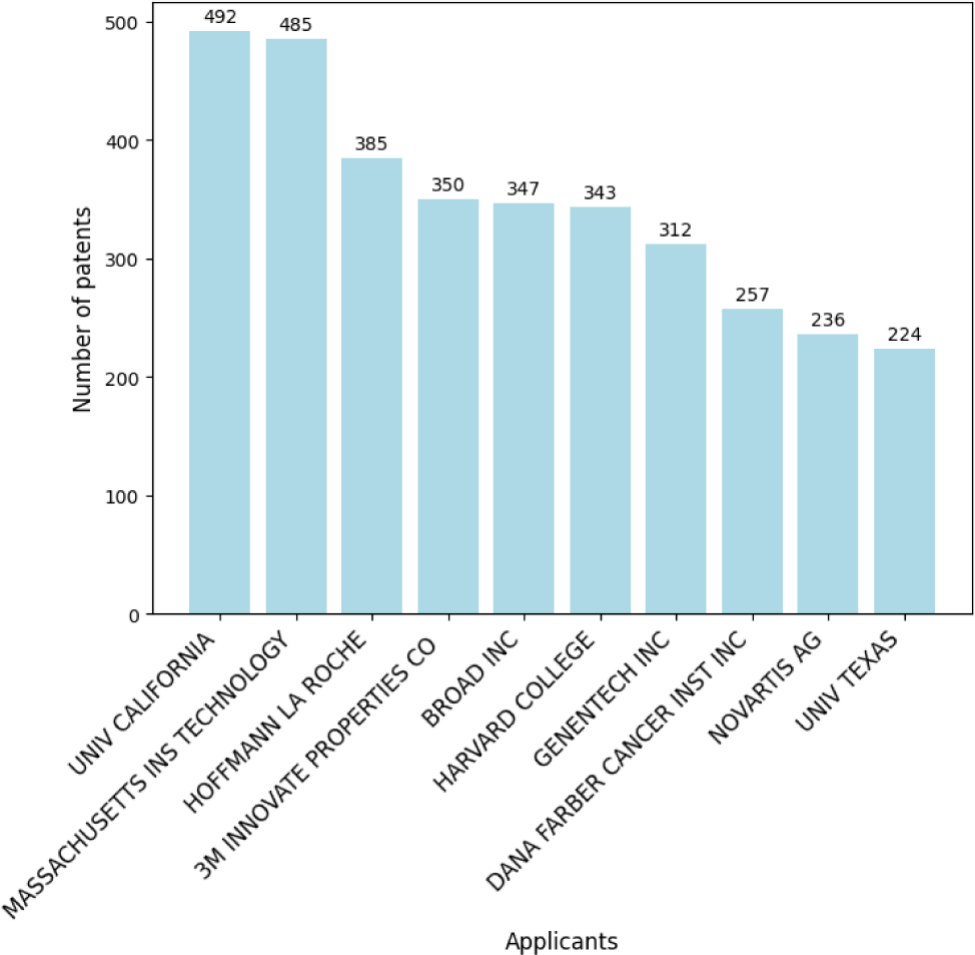
Top 10 patent applicants in nanotechnology.

**Figure 18:**
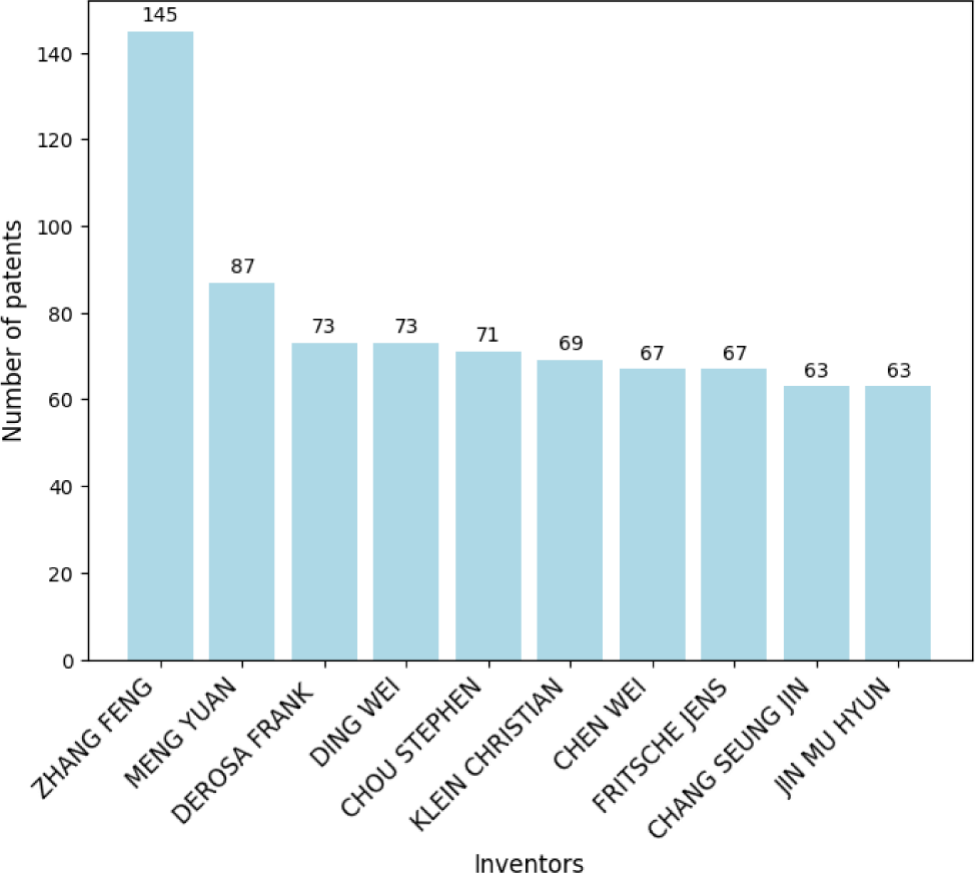
Top 10 inventors of nanotechnology-related patents.

Beyond academic research, this field also has a significant economic impact. Two searches were conducted in the ESPACENET database. The first search used the following keyword combination: ((vitreous OR eye OR glaucoma) AND (nanoparticles OR nanoemulsions OR nanocarriers OR nanoliposomes)), filtered to cover the last 15 years (2009–2024). This search yielded 43 118 patents. Figure 15 presents the normalized distribution of patents filed in the top five countries. It shows that patents registered with WO (World Intellectual Property Organization) lead in registrations, indicating that global coverage is prevalent in this field. Figure 16 illustrates the annual distribution of patent filings, with 2021 showing a peak of 4413 registrations. Figure 17 lists the top ten patent applicants, with the University of California leading with 492 filings. Figure 18 highlights the top ten inventors by patent filings, with ZHANG FENG as the leading inventor, holding 145 patents.

### 5.2 Microtech

**Figure 19:**
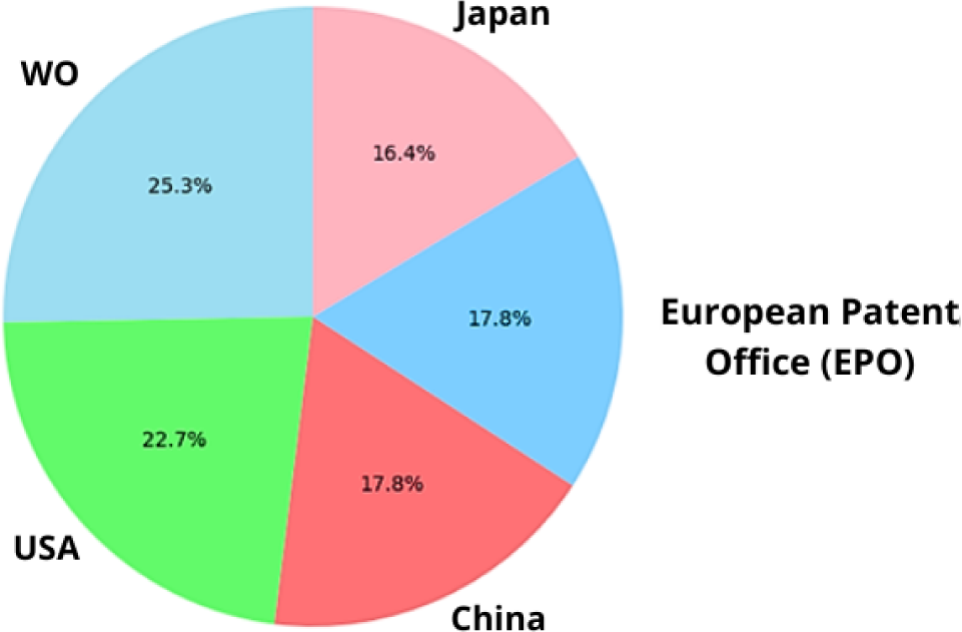
Percentage distribution of patent registrations by country in microtechnology.

**Figure 20:**
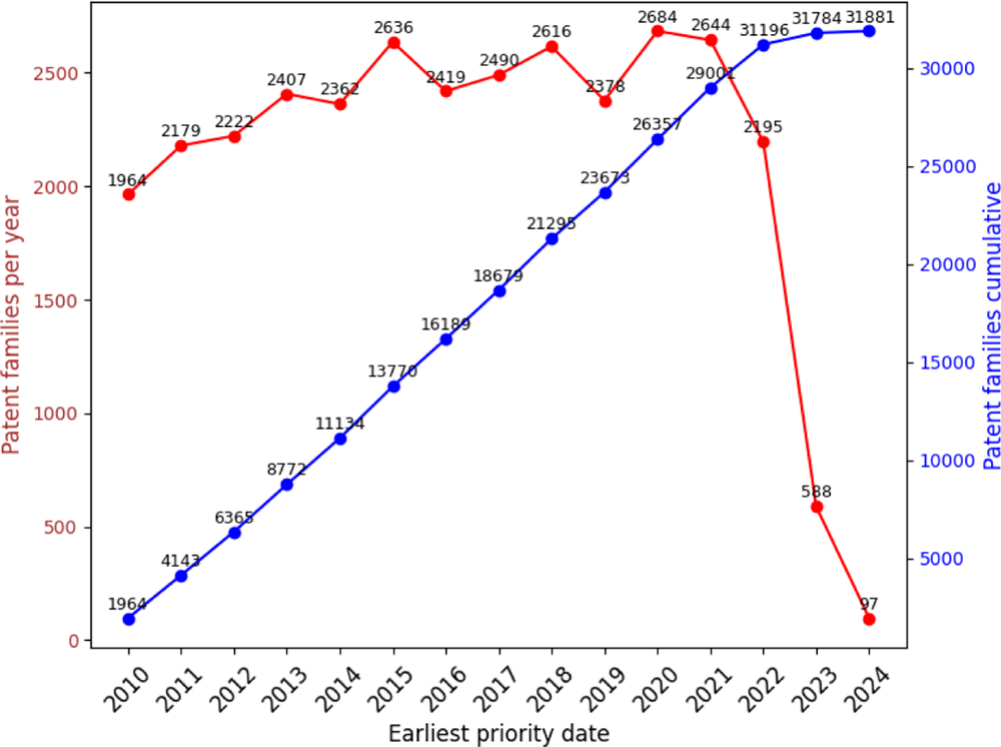
Annual and cumulative number of microtechnology patent families.

**Figure 21:**
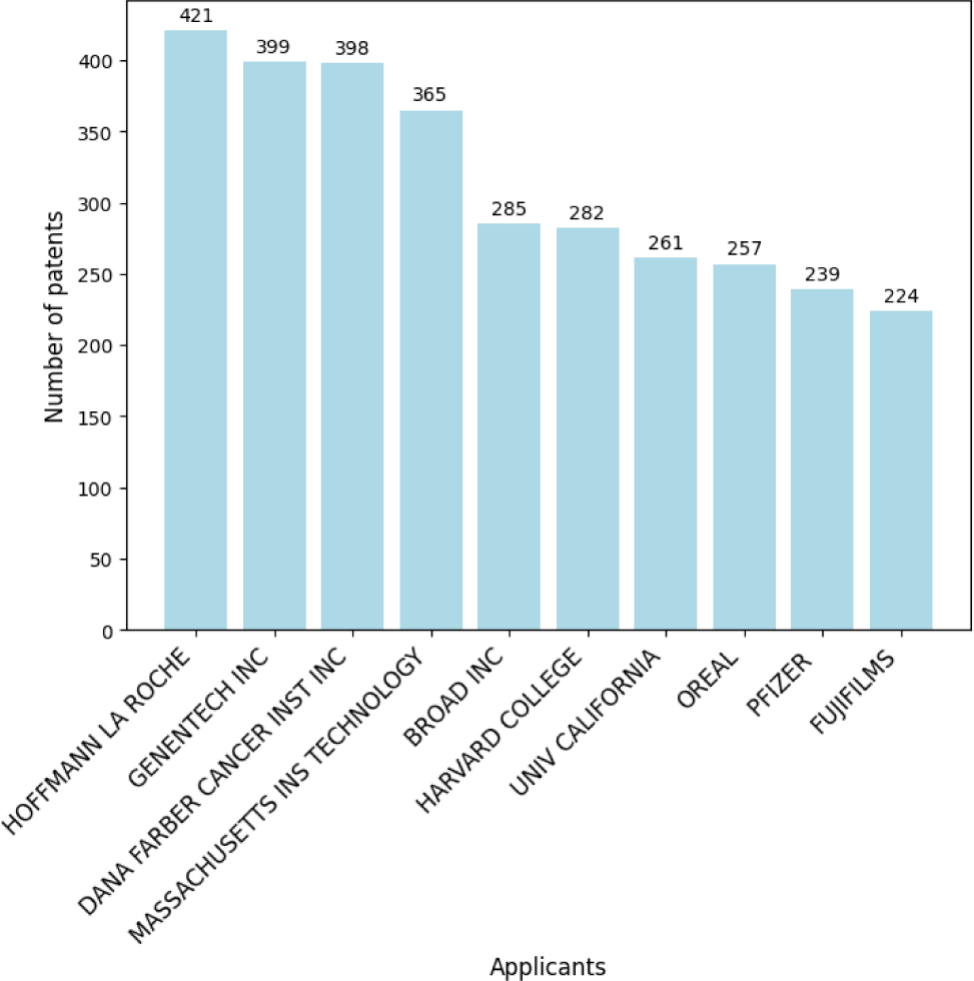
Top 10 patent applicants in microtechnology.

**Figure 22:**
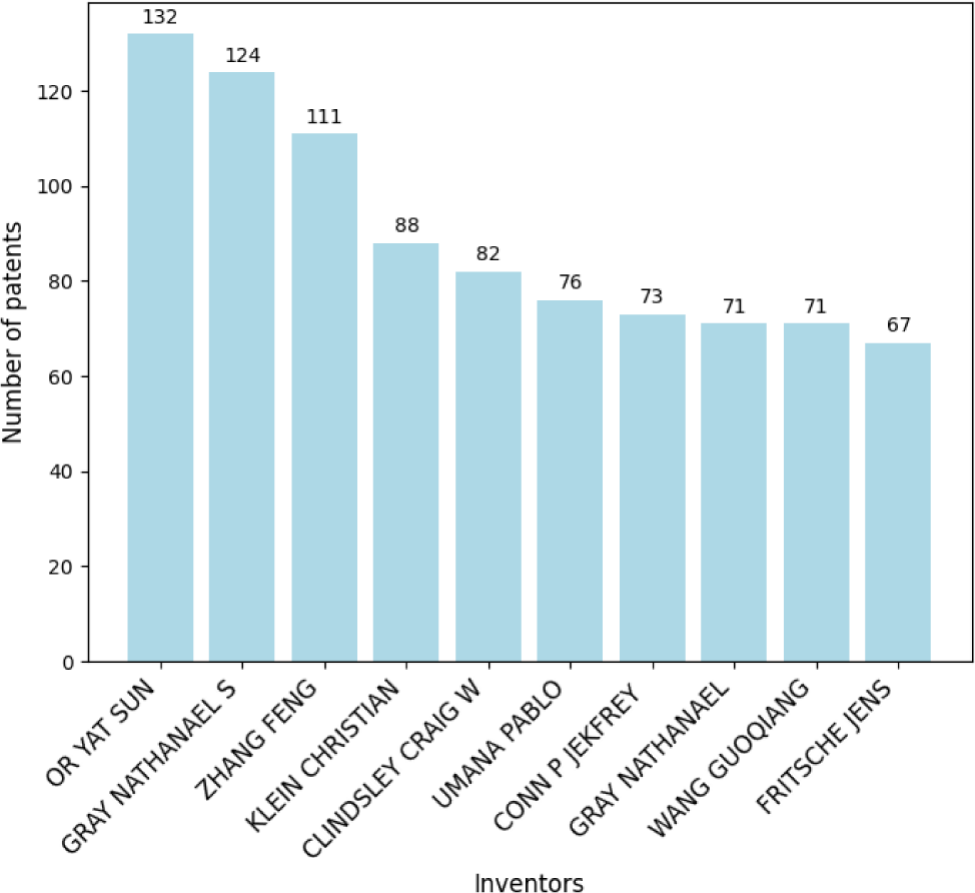
Top 10 inventors of microtechnology-related patents.

The second search used the following keyword combination: ((vitreous OR eye OR glaucoma) AND (microparticles OR microemulsions OR microneedles)), filtered to cover the last 15 years (2009–2024). This search returned 31 881 patents. Figure 19 displays the normalized distribution of patents filed in the top five countries, showing that WO (World Intellectual Property Organization) patents again lead in registrations, indicating continued global coverage in this area. Figure 20 shows the annual distribution of patent filings, peaking at 2684 registrations in 2020. Figure 21 lists the top ten patent applicants, with Hoffmann-La Roche leading with 421 filings. Figure 22 highlights the top ten inventors by patent filings, with OR YAT SUN as the leading inventor, holding 131 patents.

## 6 Opportunities, Challenges, and Prospects

Advancements in micro- and nanotechnology hold immense potential to revolutionize the treatment of eye diseases, particularly glaucoma. With the development of nanoparticles, nanorobots, and microrelease devices, new treatment methods offering greater precision and effectiveness are emerging. For instance, controlled drug release directly into ocular tissue marks a significant improvement over conventional eye drops, which often suffer from low bioavailability and rapid elimination. These innovations enable more targeted, prolonged, and less invasive treatments, greatly enhancing clinical efficacy and reducing side effects.

One key opportunity lies in integrating these technologies into established medical practices. Devices such as therapeutic contact lenses and biodegradable implants have demonstrated the ability to prolong drug delivery, reduce administration frequency, and minimize discomfort associated with ongoing eye treatments. Coupled with nanoparticle technologies such as polymer- and liposome-based systems, these devices could improve patient adherence and yield better therapeutic outcomes. In the future, combining advanced release systems with sensors that monitor eye conditions in real time could transform eye disease management. Additionally, methodological advancements in synthesis and encapsulation offer valuable insights for academic research.

Despite these promising opportunities, there are considerable challenges in achieving large-scale clinical adoption. Issues such as biocompatibility, long-term safety, and mass production of nanotechnology devices remain significant barriers. Extensive preclinical and clinical testing is required to ensure that these technologies are safe and effective across diverse patient populations. Furthermore, regulatory approval processes may present additional obstacles to commercializing these innovations.

Addressing these challenges will require increased interdisciplinary collaboration among engineers, materials scientists, physicians, and public health experts. Such cooperation is essential for overcoming technical barriers and bringing these innovations into clinical practice. Ultimately, micro- and nanotechnology hold the potential to radically transform the treatment of eye diseases, offering more effective, personalized, and accessible solutions to millions of patients worldwide.

## 7 Conclusion

The bibliometric analysis revealed a steady growth in publications within this field. The *International Journal of Pharmaceutics* emerged as the most relevant journal in terms of both publications and citations. Nominally, China emerges as the most prolific country, excelling in production volume, citation count, institutional connections, and author influence. However, when normalized by population size, England surpasses China, taking the lead in scientific production and citation impact. The analysis also indicated that nanotechnology-based devices are more prominent than microtechnology-based alternatives, likely due to their versatile applications, underscoring the revolutionary potential of these approaches in enhancing the efficacy of ocular treatments. Innovations such as therapeutic contact lenses and microneedles further highlight the promise of less invasive therapies. Additionally, various pharmacological options for reducing intraocular pressure were identified, from agents that inhibit aqueous humor production and activate enzymes to dilate the trabecular meshwork to neuroprotectants to delay optic nerve degeneration.

Through bibliometric analysis, this study demonstrates that emerging technologies, such as nanoparticles and micro- and nanotechnology systems, can potentially transform eye disease treatment, particularly for glaucoma. The findings reinforce the importance of these technologies in improving therapeutic efficacy and reducing side effects, while pointing toward future trends in treatment personalization.

Despite significant advances, substantial challenges remain for widespread adoption, including biocompatibility, scalability, and long-term safety. Developing more efficient synthesis methodologies and conducting comprehensive clinical trials will be essential for the transition of these innovations into clinical practice.
